# The Role of Communities in Mental Health Care in Low- and Middle-Income Countries: A Meta-Review of Components and Competencies

**DOI:** 10.3390/ijerph15061279

**Published:** 2018-06-16

**Authors:** Brandon A. Kohrt, Laura Asher, Anvita Bhardwaj, Mina Fazel, Mark J. D. Jordans, Byamah B. Mutamba, Abhijit Nadkarni, Gloria A. Pedersen, Daisy R. Singla, Vikram Patel

**Affiliations:** 1 Division of Global Mental Health, Department of Psychiatry and Behavioral Sciences, The George Washington University, Washington, DC 20037, USA; abhardwa@alumni.unc.edu (A.B.); gapedersen@gwu.edu (G.A.P.); 2 Division of Epidemiology and Public Health, University of Nottingham, NG7 2RD, UK; laura.asher@nottingham.ac.uk; 3 Department of Psychiatry, University of Oxford, Warneford Lane, OX1 2JD, UK; mina.fazel@psych.ox.ac.uk; 4 Center for Global Mental Health, Institute of Psychiatry, Psychology and Neuroscience, King’s College London, London SE5 8AF, UK; mark.jordans@kcl.ac.uk (M.J.D.J.); abhijit.nadkarni@kcl.ac.uk (A.N.); 5 War Child, Research and Development, 1098 LE, Amsterdam, The Netherlands; 6 Butabika National Mental Hospital, 2 Kirombe-Butabika Road, P.O. Box 7017 Kampala, Uganda; byamamutamba@yahoo.com; 7 YouBelong, P.O. Box 36510 Kampala, Uganda; 8 Sangath, Socorro, Porvorim, Goa 403501, India; 9 Department of Psychiatry, Sinai Health System & University of Toronto, Toronto, ON M5G 1X5, Canada; daisy.singla@utoronto.ca; 10 Department of Global Health and Social Medicine, Harvard Medical School, Harvard University, Boston, MA 02115, USA; vikram_patel@hms.harvard.edu; 11 Harvard T. H. Chan School of Public Health, Harvard University, Boston, MA 02115, USA

**Keywords:** community, global health, low- and middle-income countries, mental disorders, meta-review, paraprofessionals, psychological treatments

## Abstract

Community-based mental health services are emphasized in the World Health Organization’s *Mental Health Action Plan*, the World Bank’s *Disease Control Priorities*, and the Action Plan of the World Psychiatric Association. There is increasing evidence for effectiveness of mental health interventions delivered by non-specialists in community platforms in low- and middle-income countries (LMIC). However, the role of community components has yet to be summarized. Our objective was to map community interventions in LMIC, identify competencies for community-based providers, and highlight research gaps. Using a review-of-reviews strategy, we identified 23 reviews for the narrative synthesis. Motivations to employ community components included greater accessibility and acceptability compared to healthcare facilities, greater clinical effectiveness through ongoing contact and use of trusted local providers, family involvement, and economic benefits. Locations included homes, schools, and refugee camps, as well as technology-aided delivery. Activities included awareness raising, psychoeducation, skills training, rehabilitation, and psychological treatments. There was substantial variation in the degree to which community components were integrated with primary care services. Addressing gaps in current practice will require assuring collaboration with service users, utilizing implementation science methods, creating tools to facilitate community services and evaluate competencies of providers, and developing standardized reporting for community-based programs.

## 1. Introduction

The incidence, prevalence, and prognosis of mental disorders is strongly linked to community-level factors [1]. The availability and integration of mental health services into communities can promote accessibility, acceptability, affordability, and scalability of services, as well as promote adherence to treatment and increase the likelihood of positive clinical outcomes [2,3,4]. Moreover, community services can play a crucial role in promoting mental health awareness, reducing stigma and discrimination, supporting recovery and social inclusion, and preventing mental disorders [5,6,7]. It follows that international action plans and guidelines emphasize community mental health care. The World Health Organization’s (WHO) *Mental Health Action Plan for 2013–2020* calls for the provision of comprehensive, integrated mental health and social care including promotion and prevention programs in communities integrating the perspectives and engagement of service users and families [8]. The WHO *QualityRights Toolkit* requires the establishment of community-based, recovery-oriented services [9]. Article 19 of United Nations *Convention on the Rights of Persons with Disabilities* asserts that persons with disabilities, including psychosocial disabilities, should be provided with support to live independently in the community [10].

The World Psychiatric Association’s (WPA) guidance on community mental health care characterizes community-oriented care as having a population and public health focus, community-based case finding, services available within half a day’s travel, participatory decision making, self-help and peer support for service users, treatment initiation in primary care facilities and communities, stepped care, specialist supervision, collaboration with non-governmental organizations (NGOs), and networks across services, communities, and traditional and religious healers [11,12]. Similarly, the third edition of the World Bank’s *Disease Control Priorities* recommends community platforms for the following mental health service components: training gatekeepers for early identification and delivery of low-intensity psychosocial support, establishing peer and family support groups, raising awareness about harmful substance use, implementing workplace stress reduction programs, supporting community-based rehabilitation, and establishing community programs for child and adolescent mental disorders such as parenting programs with special attention to early childhood enrichment and life skills for adolescents [13].

The importance of community mental health care may have even greater relevance in low- and middle-income countries (LMIC) compared to high-income countries (HICs). The gap between the burden of mental disorders and available evidence-based services is staggering in LMIC. Whereas one out of five persons with depression receive minimally adequate care in HICs, only one out of 10 receive care in upper-middle income countries, and one out of 27 in lower-middle income countries [14]. The picture has been equally bleak for severe mental disorders, where it is not uncommon for persons with schizophrenia to spend more than five years with active psychosis before evidence-based treatment is initiated [15]. In community platforms, such as the home, prayer camps, traditional healing centers, and other religious institutions, persons with severe mental illness may be chained, kept in forced seclusion, and suffer sexual abuse and other forms of exploitation [16,17,18]. Therefore, outreach to communities is crucial for human rights protection. Standard facility-based psychiatric services are often inadequate to address negative social determinants of mental health such as economic deprivation, ethnic/racial discrimination, exposure to traumatic events, and violation of human rights [16,19,20]. Moreover, the high levels of stigma toward people with mental illness among the general public and health workers is a barrier to seeking specialized mental health services [21]. Additional constraints include lack of evidence on screening and detection programs to identify persons needing care, lack of transportation infrastructure to reach health facilities, shortage of health personnel trained in mental health care, and lack of psychological treatments at health facilities [22,23,24]. Given these challenges, it is vital to determine how to effectively work in communities and with community-based service providers for mental health care delivery in low-resource settings.

The primary objective of this review is to map the landscape of evidence-based community components of mental health interventions and draw lessons about their implementation. We characterize community components as any aspects of mental health intervention delivered outside of a primary care or specialty mental health care setting. Although the WPA defines community mental health care more broadly than simply the setting in which a program is delivered, we pragmatically focus on the activities and implementation of mental health services in communities. This is crucial to identifying the key competencies for providers so that governments and organizations can scale up these programs and explore new avenues for community-based care. Additionally, it is important to identify gaps in practice and knowledge so that research can evaluate the effectiveness of community-based strategies.

## 2. Materials and Methods

### 2.1. Search Strategy

We conducted a review of reviews in November 2017. Our review question was “What interventions have been evaluated in community settings for treatment and prevention of mental disorders in low- and middle-income countries?” We searched three databases: OVID MEDLINE/PubMed (since 1946), PsycINFO (since 1806), and Web of Science (Social Science Citation Index). Search headings included ‘developing countries/’ or ‘global health/’, ‘mental health/’ or ‘mental disorders/’. We identified additional sources through our prior participation in systematic reviews and bibliography scans of included references. We limited results to publications indexed as review articles in the search engines. We registered protocol in PROSPERO, registration number: CRD42018092014. 

### 2.2. Eligibility Criteria

We included studies if they were reviews of randomized and non-randomized controlled trials for psychiatric conditions in LMIC. We extracted community components of interventions with ‘community components’ operationalized as aspects of mental health interventions not conducted in health facilities (i.e., services delivered within primary care clinics, specialty services, and psychiatric hospitals were excluded). ‘Intervention’ refers to any activity deliberately administered to promote recovery or remission of a mental disorder, reduction of symptoms, or improvement of functioning among persons living with a mental disorder. We excluded intervention programs that were delivered exclusively within primary care or health facilities. For interventions in which some components were conducted in a community platform and others in a health facility, we extracted information related to those implemented in the community. For this review, we considered a community health worker or lay health worker as a community provider, whereas primary care workers were not community providers. Within each review, we only extracted information based on the specific studies that included a community component. Therefore, not all studies in a review were extracted for content analysis. For the extraction, we use the term ‘platform’ to refer to the setting where the interventions occur, e.g., schools, homes, public spaces, and community buildings.

Eligible psychiatric conditions were based on five broad diagnostic categories: common mental disorders, perinatal mental disorders, psychotic disorders, substance use disorders, and child and adolescent mental disorders. LMIC status was determined by World Bank criteria for low- or middle-income economies at the time of the intervention trial [25]. We only included systematic reviews. We excluded reviews if they did not summarize mental health intervention trials, if the trials were not conducted in LMIC, and if quantitative outcomes reporting effectiveness were not presented. If a review contained both HIC and LMIC studies, we included the review but only extracted information on the LMIC studies. If all studies and descriptive information in a review were reported in a more recent updated review, then the earlier review was excluded.

### 2.3. Data Extraction and Synthesis

We extracted five domains for each review based on criteria adapted from a prior review of psychological treatments in LMIC [26]:

- **Domain 1. WHY** was a community component or community platform selected for the intervention or an aspect of the intervention? What was the rationale for how the community component would facilitate achievement of the intervention objectives?
- **Domain 2. WHERE** was the community component conducted or delivered? What were the barriers or facilitators in that setting, i.e., platform?
- **Domain 3. WHAT** were the community components of the intervention? This could include specific strategies (e.g., community sensitization, awareness raising), or specific therapies (e.g., behavioral activation, cognitive behavioral therapy).
- **Domain 4. WHO** is the delivery agent for the community component? How were they recruited, trained, supervised, and certified, and how was their competency determined?
- **Domain 5. HOW** were the community components implemented? This refers to any descriptions of the process by which the actions are implemented, including roles of specialist mental health workers and other support staff, specific manuals, technologies, and tools.

In addition to the above a priori extraction fields, after preliminary coding, we added ‘**Harms and Risks**’ as an extraction field given the importance of this issue for ethical, safe, and effective community programs. We include harms and risks both within the context of persons receiving mental health services and for persons delivering community components.

Two authors (B.A.K. and A.B.) examined all reviews for eligibility. Eligible reviews were divided among the authors, with reviews assigned based on the authors’ expertise in specific psychiatric conditions. The authors then extracted data as summaries, from their allocated reviews for the five data domains described above. Some of the authors had participated in some of the reviews eligible for this meta-review. Therefore, when possible, they consulted their original data extraction tools to supplement their summaries with information not reported in the final review publications. When authors encountered ambiguous findings or descriptions, a second author was consulted to make extraction recommendations. Analysis was limited to narrative summaries. Given heterogeneity in study design and differential coding and extraction strategies used in the reviews, a meta-analysis was not conducted.

### 2.4. Quality Assessment of Included Studies

The quality assessment was performed using the AMSTAR 2 criteria for systematic reviews of randomized and non-randomized studies of health care interventions. By going through the key sequential steps of conducting a systematic review, the AMSTAR 2 assesses both quality of the systematic review along with identifying any critical weaknesses [27]. Two authors (B.A.K. and A.B.) scored each article on the 16 quality criteria.

## 3. Results

### 3.1. Search Results and Review Characteristics

**Figure 1:**
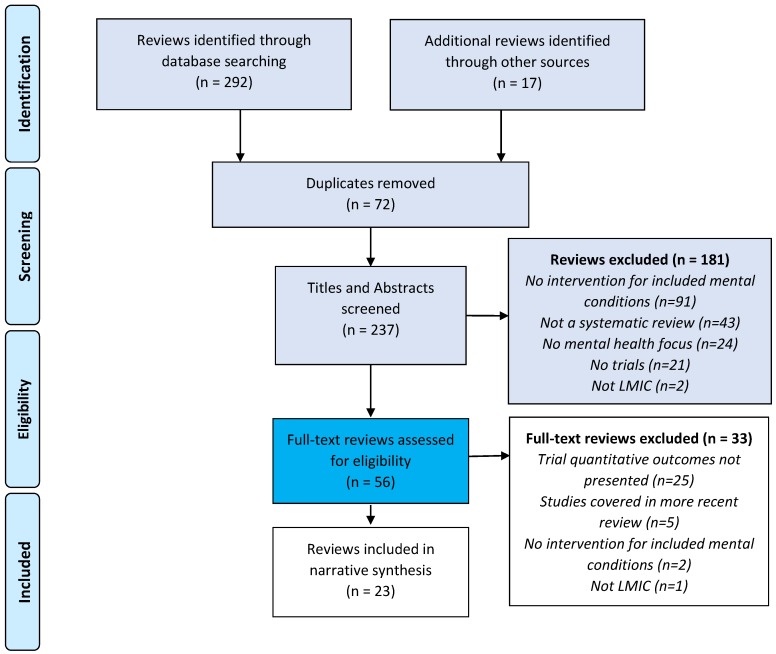
Preferred Reporting Items for Systematic Reviews and Meta-Analyses (PRISMA) Diagram.

From our database searches, we identified 292 reviews plus an additional 17 reviews from other sources. Twenty-three reviews met eligibility criteria for the narrative synthesis (Figure 1). Of these, ten reviews covered common mental disorders, eight reviews covered perinatal mental disorders, four reviews addressed psychotic disorders, two reviews covered substance abuse disorders, and nine reviews covered child and adolescent mental health. Some reviews covered multiple conditions (e.g., common mental disorders, substance abuse, and child and adolescent mental disorders; see Table 1).

Of the ten eligible reviews identified for *common mental disorders*, we included eight individual each of which had a community-based component and a positive outcome in a randomized controlled trial (RCT) [26,29,31,33,35,36,43,44,45,49]. The predominant reason for excluding common mental disorder studies was that all activities in a trial took place within a primary care facility. The countries for the eight studies were Brazil [50], China [51], Colombia [52], the Democratic Republic of Congo (DRC) [53], Pakistan [54], Sri Lanka [55], Thailand [56], and Uganda [57].

For *perinatal mental disorders*, we included 19 studies from 17 publications. We excluded studies that did not target mothers or those without a psychological component targeting mental health (e.g., psychosocial stimulation programs only). The majority of studies were implemented in China (n = 5) [58,59,60,61,62] and sub-Saharan Africa (n = 4) [63,64,65,66], followed by South Asia (n = 4) [54,67,68,69], Mexico and Chile (n = 3) [70,71,72], and Eastern Europe (n = 1) [73,74].

For persons living with *psychosis*, five systematic reviews were found relating to community-based interventions [28,36,38,45,49], from which 13 relevant evaluations were identified [75,76,77,78,79,80,81,82,83,84,85,86,87]. All eligible evaluations were set in middle-income countries, including six in China [76,81,82,85,86,87], three in India [77,78,80], two in Iran [79,83] and one each in South Africa [75] and Turkey [84].

For *substance use disorders*, two reviews met eligibility for this review, with one review summarizing four studies and another summarizing two studies [47,49]. From one substance use disorder review [47], all four studies met eligibility criteria, two from Mexico [88,89], one from Vietnam [90] and one from Malaysia [91]. The two studies using non-specialists for treatment of substance use disorder in the van Ginneken and colleagues Cochrane review [49] were excluded because they both took place in a clinical setting.

For *mental disorders affecting children and adolescents*, nine reviews included information on interventions in 122 studies. For three of the reviews [37,41,48], most studies were on non-selected populations of children, therefore specific disorders were rarely targeted and the interventions were more aligned with mental health promotion and prevention. Two of the reviews focused on children and adolescents affected by war and other armed conflicts [39,40]. One review evaluated the context in which different intervention approaches worked in LMIC covering 54 trials [42]. Two other reviews focused on promotion/prevention interventions [30,44].

### 3.2. Quality Assessment of the Included Reviews

The reviews were generally of high quality, meeting the most reporting requirements of AMSTAR 2 (Table 2). The domains in which the reviews performed poorly on quality criteria were reporting the funding for each individual study (4% of the reviews) and exclusion justification (26% of the reviews). The only study meeting all AMSTAR quality criteria was the Cochrane review completed by van Ginneken and colleagues [49].

### 3.3. Domain 1. Why Are Community Components Selected for Mental Health Interventions?

Five key themes were identified: (1) The majority of studies reported that community platforms were an alternative to primary care to enhance the reach of services; (2) community components could augment clinical services, such as enhancing medication adherence; (3) community programs were also implemented to increase the likelihood of family involvement, which would in turn improve quality of life, functioning, and inclusion; (4) community platforms also had economic benefits not observed in primary care and specialty settings; and (5) community platforms used to promote social inclusion.

#### 3.3.1. Community Platforms as an Alternative to Primary Care

Delivering care that was easily accessible to persons with mental illness was a reason for community-based services in all reviews. Poor transportation infrastructure and lack of patients’ economic resources to afford transportation necessitated community-based delivery [26,44,49]. Two Indian studies highlighted the need to improve accessibility of treatment for persons with psychosis, particularly in rural areas or where transport costs to outpatient clinics may be prohibitive [78,80].

Cultural barriers to utilizing primary-care based services were reported: for women with common mental disorders in Pakistan, cultural behavioral norms requiring a male relative when leaving the house impacted accessing primary care facilities and motivated the use of home visits by lay female counselors [54]. In regions affected by armed conflict, e.g., Uganda and the Democratic Republic of Congo, primary care services were either non-functional or controlled by threatening political groups [53,56,57]. School-based services provided care that was accessible to all students [37].

#### 3.3.2. Enhancing Quality of and Engagement with Clinical Care

Some community components enabled greater exposure to clinical services than would have been possible through primary care interventions. Among depressed patients in Sri Lanka who attempted suicide, a brief mobile treatment including face-to-face engagement, telephone calls, and encouraging text messages was used; this was done because of the hypothesized benefit for continued engagement to monitor suicide risk and intervene early [55]. The authors expected that primary-care services in isolation would have been inadequate to achieve the intended clinical benefits for depression and suicide risk reduction. Another reason for delivery outside the health facility was that care delivered in the home was thought to facilitate treatment adherence during a sensitive life transition, particularly for mothers during the antenatal and postnatal period [34]. Intervention developers hypothesized that community-based providers inspired a greater therapeutic alliance, e.g., non-specialist providers from the same community facilitated trust between provider and beneficiary and increased likelihood of sustainability [26,46]. In a community-based rehabilitation program for persons with schizophrenia in India, lay health workers were better able to communicate with the local community because they used local cultural idioms leading to more effective psychosocial support [78]. For persons with psychosis, interventions were largely concerned with reducing inappropriate hospitalizations, and these studies tended to be set in better resourced urban settings such as cities in China, Iran, and South Africa [75,79,83,86].

#### 3.3.3. Involvement of Family Members

Most evaluations set in China highlighted that family members are the main source of care for people with psychosis, which necessitated family involvement for successful interventions [81,82,85,86,87]. Overall, all interventions for psychosis, except one in South Africa [75], explicitly referred to family involvement in the service delivery. Another study in China for mood disorders included promoting family involvement as a central component of the intervention design [51]. Similarly, for perinatal disorders, conducting the intervention in the women’s home made it possible to engage the whole family who shared the responsibility of taking care of the newborn [46].

#### 3.3.4. Economic Productivity

Economic benefits, including both economic productivity of families and cost-effectiveness of community delivery, were also cited. The family-support approach for discharged patients with depression in China showed significant financial and time productivity benefits for family members [51]. In Uganda, the community-based group interpersonal psychotherapy for depression benefited women’s economic productivity (but not men’s economic status) [57]. In Colombia, asynchronous telepsychiatry via primary care workers in prisons was more cost effective than synchronous telepsychiatry with psychiatrists [52].

#### 3.3.5. Social Inclusion

A minority of interventions for persons with psychosis mentioned the potential for community engagement to benefit their recipients in terms of changing attitudes and creating a support network for homeless individuals in rural China [85], and supporting social inclusion and facilitating livelihood opportunities or rehabilitation in rural India [77,78]. For children and adolescents, school-based services were considered to promote social inclusion because the setting was less stigmatizing than visiting a mental health care facility [37].

### 3.4. Domain 2. Where Are Community Components Delivered?

Community platforms included (1) homes, (2) schools, and (3) settings such as nongovernmental organization offices, prison, and community centers. There was also increasing use of (4) technological platforms ranging from telephone calls to tele-psychiatry services in communites. 

#### 3.4.1. Homes

For common mental disorders, one study involved non-specialists trained to do home visits for women with depression in Pakistan [54]. For perinatal interventions, most programs were implemented in the participants’ home environment (n = 8) in Chile, Mexico, Pakistan, South Africa, and Turkey [63,64,67,68,70,71,72,74]. For persons with psychosis, psychosocial components were delivered solely in the participants’ home in six studies in China, India, and Iran [77,78,80,82,83,85]. 

#### 3.4.2. Schools

For children and adolescents of school-going age, successful interventions were delivered in schools (n = 19), compared to other community spaces (n = 6) or homes (n = 3). In one of the armed conflict reviews, interventions were school-based (n = 12), followed by interventions within refugee or internally-displaced persons camps (n = 5), and homes (n = 3) [42]. Similarly, in the other armed conflict review, seven interventions were reported to utilize the school platform, one in the home, and six within the community [40]. See Box 1 for a description of the Classroom-Based Intervention.

Box 1 **Case Study of Community Based Mental Health Care for Children and Adolescents in Humanitarian Settings: Classroom Based Intervention.**

A community program with a school-based component was implemented in five conflict- affected countries: Sri Lanka, Burundi, South Sudan, Indonesia and Nepal [92,93,94,95,96,97,98,99,100].

**Why:** Schools were considered to increase access to children and reduced risks of stigmatization. The school-based approach increased community and parental acceptance and buy-in.

**Where:** The essential delivery platform of this program was in or around the school and schoolyard. The main barrier to using the school as the primary platform for implementation was that it was hard to include non-school going children.

**What:** This manualized intervention consists of 15 sessions delivered over five weeks in 90-minute sessions. Contents include cognitive behavioral techniques, psychoeducation, strengthening coping, guided exposure to past traumatic events through drawing, cooperative games, structured movement, music, drama, and dance. Each group included approximately 15 children. Children with sustained and serious problems after termination of the Classroom-Based Intervention (CBI) were assessed and referred to the next tier of the system, which was counseling. Counselors were responsible for providing problem solving therapy for children individually or including their families. The average number of sessions ranged from 2.2 to 7.5 depending on the country.

**Who:** Local and psychosocial workers, counselors, and teachers who were not mental health professionals implemented the program. CBI facilitators were trained for two weeks. Counselors were trained for one month, except in Nepal where they were trained for four months. The CBI intervention was delivered by trained facilitators, always with two co-facilitating the sessions. In some countries (Sri Lanka, Burundi), one of the co-facilitators was a teacher. This had both advantages and disadvantages. The advantages were that teachers usually stayed in the community and could integrate the learning of new skills into daily teaching practice. The disadvantages were that the teachers have already established relationships with children, often based on positions of authority, making it difficult to take on the position of a playful CBI facilitator. Bi-weekly supervision by a psychologist or senior counselor was provided for both the facilitators of CBI and the counselors providing treatment for children with more severe problems.

**How:** The program included school-based primary screening. The screener was developed for conflict-affected settings and used a template with context specific components adapted within each country. Before the screening, community sensitization was done through community meetings with stakeholders to increase engagement of the community with the program. In Burundi radio programs were used for the awareness raising. There were some small-scale initiatives within the programs where children were involved in delivering the intervention. For example, in Burundi, children were involved in a ‘child-to-child’ initiative where children identified other children in their communities that needed social or financial support, which was subsequently organized by the children.

#### 3.4.3. Other Community Platforms

Non-governmental organizations working in settings of political violence delivered group-based interventions for common mental disorders in their offices or other community platforms in the Democratic Republic of Congo and Uganda [53,57]. For persons with psychosis, only in the community-based rehabilitation evaluation in rural India was an intervention component, village health groups, located in a community location outside of the participants’ home [78]. All studies included in one of the reviews for substance use disorders [47] were based in community centers providing deaddiction services. The target population included spouses and adult family members of drug/alcohol users and abusers. In one study, the care was delivered for adults in a prison in Colombia [52].

#### 3.4.4. Technology and Digital Platforms

Increasingly, technology is being employed to increase access to care as well as personalization of treatment [45]. For example, asynchronous telepsychiatry via primary care workers in prisons was both clinically effective and cost effective when evaluated in Colombia [52]. In Brazil, persons with depression received care through home-based video teleconferencing [50]. Both telephone calls and encouraging text message reminders were used effectively for persons with a history of depression and suicidality in Sri Lanka [55]. For perinatal mental health care five studies, all in China evaluated implementation via telephone [58,59,60,61,62]. Media, specifically radio, was used in two studies in China to promote mental health awareness [82,85].

### 3.5. Domain 3. What Are the Community Components Delivered?

Community components covered the spectrum from population-wide programs to group and individualized psychological treatments: (1) population-wide programs focused on mental health awareness-raising and reducing stigma, and mental health promotion programs were most commonly delivered to children and adolescents; (2) psychoeducation was described for all conditions, but it was especially central to the programs involving families of persons living with psychosis or substance abuse disorders; (3) skills training was reported for all conditions, with child and adolescent programs focusing on life skills; (4) case management was used for persons with psychosis; and (5) an array of psychological treatments was reported both for children and adults.

#### 3.5.1. Population and Community-Wide Mental Health Awareness Programs

Three interventions for persons with psychosis involved mental health awareness-raising, including two in China via local radio stations [82,85]. Programs for children and adolescents in armed conflict settings included community-level interventions to decrease stigma and raise awareness in Burundi, Indonesia, Nepal, Sri Lanka, and South Sudan [92]. Regarding prevention [44], non-specialist providers were mainly involved with primary more than secondary prevention initiatives. All six of the prevention programs that targeted child mental health outcomes involved primary prevention with four of the studies using indicated prevention and only two studies using selective prevention. Strategies included emotional and social support, education, and awareness-raising targeting women’s groups and caregivers [44]; other studies included microfinance, parent training and recreational activities [30]. Some child and adolescent mental health promotion programs included creativity and arts-based interventions as well as games, meditation, and yoga [37]. Other child and adolescent programs aimed to promote mental health through conflict resolution and mediation, nutritional supplementation, recreational treatments, maternal support, microfinance, and physical medical services. In settings of armed conflict, other activities included risk prevention, integration of psychosocial support in non-medical sectors, multi-sectoral coordination, strengthening ecological support systems, restoring traditional beliefs and practices, caregiver capacity building and support, and community mapping of needs and resources. In addition, adolescent-focused activities included peer dialogues, community drama, adolescent mobilization for social action, traditional-cultural ceremonies, support for reintegration and family reunification of ex-child soldiers, self-help groups, utilization of community and cultural resources, formal and non-formal education, reconciliation workshops, and child protection services [39,40].

#### 3.5.2. Psychoeducation

For adults with common mental disorders, some interventions had a psychoeducation component [26]. Perinatal programs included individual and family psychoeducation, typically delivered in the home [32,46]. All home-based interventions for persons with psychosis included psychoeducation with information about symptoms, illness course, treatment, and relapse prevention. Some interventions included education about treatment and adherence support contributing to continuation of anti-psychotic medication [76,77,78,79,81,82,85,86,87]. A program in Malaysia for families affected by substance use disorders included family psychoeducation, support groups, family retreats designed to elicit resilience and healing within the family [91]. Psychoeducation was commonly used for children and their parents and teachers. For the general population, psychoeducation about child mental health was delivered in open information sessions with key stakeholders assisting in increasing engagement with interventions [37]. For settings with children affected by armed conflict, community sensitization and public awareness programs were conducted [39]. Tier 1 of a stepped care program for children affected by armed conflict in Bosnia included school-based psychoeducation and skills training [101].

#### 3.5.3. Skills Training and Community-Based Psychosocial Rehabilitation

For perinatal mental disorders, most programs had parenting skills training [32,34,46]. Parent training included supporting secure infant attachment, training on childhood development, and promoting parental responsiveness. Psychosocial interventions for persons with psychosis were multi-faceted and focused on rehabilitation. Components included family activities, social and independent living skills’ training, medication adherence support, and dealing with stigma [76,77,78,82,86]. A minority of studies described attempts to support livelihood or vocational activities [77,78,84,86] though this was generally only information and advice rather than arrangement of work placements. In six studies, in South Africa, India, Iran and Turkey, individuals living with psychosis were supported to access community resources and organizations including legal benefits, employment opportunities, self-help groups and other informal care networks [75,77,79,83,84,85,102]. In a program in rural India, community-based rehabilitation comprised village health groups formed of family members and key community members; these groups aimed to reduce social exclusion and support recovery by jointly planning rehabilitation activities [78]. For childhood developmental disorders, community-based rehabilitation was common [42]. This comprised building children’s skills in activities of daily living and assisting parents to find income generating activities. Life skills programs, apprenticeships, vocational skills training, income generation, and livelihood programs were often also included for children affected by armed conflict [39].

#### 3.5.4. Case Management

Some interventions for persons with psychosis included community-based case management. Integrated treatment models included assertive community treatment in South Africa [75], home-based aftercare services in Iran [79,83], and optimal case management in Turkey [84]. Though a few of these studies included components such as psychoeducation and social skills training, the overall emphasis was on supporting engagement with care following discharge from inpatient facilities. These trials were based in urban areas in upper-middle income countries. For persons living with psychosis, some interventions specified a crisis intervention component [75,82,84,86], typically activated if the individual became suicidal or aggressive.

#### 3.5.5. Psychological Treatments

For common mental disorders, group and individual psychological treatments were delivered including interpersonal psychotherapy (IPT), common elements treatment approach (CETA), cognitive processing therapy (CPT), basic techniques of cognitive-behavioral therapy (CBT), problem-solving therapy, and family psychotherapy [26]. For perinatal mental disorders, the majority of programs focused on problem-solving and behavioral activation [32,46] as well as coping with stress and interpersonal effectiveness [26,32]. One Indian study provided cognitive retraining alongside psychoeducation for persons living with psychosis [80]. Several interventions were guided by intervention manuals and/or following predetermined modules or tasks, and in some cases the cultural specificity of the content was mentioned [76,77,79,80,84].

Psychological treatments for alcohol use disorders were manualized interventions that included multiple components: ‘Intervention V’ in Vietnam [90], and Rational Emotive Behavior Therapy based coping enhancement (REBT) [88] and the 5-Step Method [89], both implemented in Mexico. The components of Intervention V targeted developing healthy family routines, care-giving with an aim to overcome family challenges, managing negative emotions, learning coping skills, developing realistic goals, and supporting positive behavior change. In REBT, the focus was correction of cognitive bias and defective information, emotional regulation strategies, assertive interpersonal skills, promotion of self-esteem, deep diaphragmatic breathing, and progressive muscle relaxation. The 5-Step Method included listening to and exploring the family’s experiences, providing relevant information, identifying coping strategies, exploring support available, and referral to specialized sources of help, if necessary.

For children and adolescents, psychological treatments included generic counselling, classroom-based interventions, and trauma-focused treatments with an emphasis on the verbal processing of past experiences. For adolescents with common mental disorders, the most effective interventions were community group treatments (e.g., using IPT). For children with severe symptoms of posttraumatic stress disorder (PTSD) in Bosnia, a program included different tiers of community-based intervention with a manualized 17-session psychological treatment at Tier 2 [101]. Other trauma approaches included school and community-based group trauma-focused CBT (n = 3) [40]. A number of school based mental health promotion activities were implemented in countries affected by conflict, with most incorporating CBT and trauma psychoeducation modules [30].

### 3.6. Domain 4. Who Delivers the Community Components of Interventions?

We categorized providers into four groups: (1) community mental health workers, (2) other health professionals, (3) formal providers outside the health system, and (4) non-formal providers (lay persons and peers). For adult common mental disorders, community health workers or non-formal providers in the community with no health or other social service roles were selected for training and care delivery. Perinatal programs were often delivered by community health workers who were already responsible for maternal and child health services. For children and adolescents, among successful studies, the majority used formal providers outside the health system (i.e., teachers, n = 9), community health workers (n = 6), other health professionals (n = 5), and non-formal providers (n = 2). For humanitarian settings, child and adolescent community components were most commonly delivered by other health professionals (counsellors, n = 13), community health workers (n = 5), peers (i.e., other students, n = 3), and non-health formal providers (social workers, n = 2, and teachers, n = 1).

#### 3.6.1. Community Health Workers

Community health workers are members of the formal health system who may be paid or unpaid. They are responsible for outreach, education, promoting adherence, and documentation and monitoring outside of the health facility. For perinatal disorders, supervised community health workers typically delivered treatment [46], such as the Lady Health Workers in Pakistan [67].

#### 3.6.2. Other Health Professionals

Nurses, primary care workers, and other health professionals who are typically facility-based have also been engaged in community mental health. In some programs, facility-based health workers without mental health specialization were trained to deliver community components. In upper middle-income countries such as Turkey and China nurses were trained to deliver treatment in the community [60,74]. For persons with psychosis, in four studies, health professionals not specialized in mental health, such as nurses, village doctors or general practitioners, were the main personnel [75,81,82,83]. A non-specialist health worker working in a prison delivered care for depression in Colombia [52], and local doctors were used in rural China [82].

#### 3.6.3. Formal Providers outside the Healthcare System

Formal providers, who have not received professional training in mental health, included teachers, law enforcement officers, and social workers. Among successful interventions for children and adolescents, the majority used teachers (n = 9). This contrasts with interventions in armed conflict settings where teachers were in the minority of providers. For prevention/promotion, of the eight child interventions delivered by non-specialist health workers, three studies used existing high school or preschool teachers. Supervision varied from intensive to regular, as was the training [49]. For behavioral disorders, school-based teacher-training was predominantly used [103].

#### 3.6.4. Non-Formal Providers

Non-formal providers are lay persons who do not have a formal role in the health or other service provision programs. Non-governmental organizations often recruit and train lay persons in the community to take on psychosocial programs. For adults with common mental disorders, local NGO workers delivered psychological treatments in Uganda, the Democratic Republic of Congo, and Thailand [53,56,57]. For psychosis, only two Indian studies by Chatterjee included interventions delivered by lay persons who had no prior role as community health workers [77,78]. Lay persons in the community were also recruited to be service providers in substance abuse treatment programs [47]. For children and adolescents, lay persons were recruited to be psychosocial workers, counsellors, caregivers, and aides. Programs were commonly delivered by lay counsellors (n = 13), followed by community workers or facilitators (n = 5), and students (n = 3). For prevention/promotion of child and adolescent mental health, the majority (five out of eight interventions) was delivered by lay persons [49]. Peers were trained for delivery of a perinatal mental health intervention in Uganda [104] (see Box 2). Substance abuse interventions were also delivered by individuals who had previously suffered from and undergone treatment for alcohol or drug abuse.

Box 2 **Case study of community based mental health care for maternal mental health.**

An example of an integrated, community-based program that targeted both maternal mental health and child development was implemented in rural Uganda [104,105]. The program was effective in improving child development and preventing maternal depressive symptoms. Additional analyses demonstrated that mothers’ perceived support from spouses and psychosocial stimulation mediated the effects of the intervention on reduced maternal depressive symptoms and improved child development scores respectively.

**Why:** Both stimulation and maternal mental health interventions have been effectively implemented by non-specialist providers (e.g., community health workers or volunteers) despite no formal training in mental health or child development. Past research showed that mother or child interactions by themselves do not appear to have sufficient effects on both mother and child. Embedding this universal program within a community platform was helpful to recruit participants, prevent stigma toward mental health, and encourage fathers to attend. Community leaders were particularly helpful in encouraging fathers to attend sessions. These approaches increased community and parental acceptance for the program.

**Where:** The peer delivery agent and participants selected the location. Thus, the program was implemented in whichever community platform was feasible and acceptable for the individual group, e.g., under a tree (weather permitting) or in a pre-school center.

**What:** The manualized program consisted of five main messages—diet, hygiene, two-way talk, play materials, and love and respect—where the message of ‘love and respect’ explicitly targeted key aspects of maternal mental health related to the mother’s relation with her child, spouse, and herself. Fathers attended specific sessions. Treatment strategies borrowed from cognitive, behavioral, and interpersonal psychotherapies, and the program itself was informed by social cognitive learning theory. The program was implemented over 12 bi-monthly sessions in group formats that took place in the community. The format involved psychoeducation, skill-building through games, Q&A sessions, and homework. Each participant received up to two home visits over the course of the program. Formats were mother-only, father-only and mother and father joint sessions. Some but not all involved bringing the child.

**Who**: Peer volunteers were trained to deliver the 12-session parenting program. Peer volunteers were selected based on their reputation in the community, interpersonal skills, and language abilities. They were trained over two weeks and supervised by experts in child health and development.

**How:** Experts were trained by the intervention developers. They were trained not only in the program materials but also in effective communication skills for groups (e.g., common skills such as empathy, a non-judgmental stance, and active listening and motivational interviewing). They were evaluated after training and during sessions by experts using a brief ‘Monitoring Form’. Process research demonstrated that both peers and expert supervisors appreciated the use of a structured form to inform program quality and feedback [105].

### 3.7. Domain 5. How Are Community Components Implemented?

Despite the emphasis for engagement with service users, there was limited description of this in the review studies. The implementation of community components typically involved community-based case finding for program participants, except for programs that recruited beneficiaries during inpatient hospitalizations. The spectrum from achieving competency, maintaining quality, and sustaining delivery entailed an array of training and supervision approaches, with a notable dearth of formal tools for competency and fidelity evaluation. Delivery formats included both group and individual, with classroom-based interventions being a specific type of group format. Some studies integrated community mental health into other programs such as maternal and child health and nutrition programs. Barriers included both cost and stigma.

#### 3.7.1. Service User Involvement in Design of Community Programs

In the review summarizing cultural adaptations for depression, the review authors noted that culturally adapted interventions tended to use stakeholder consultation [33]. For the trial in rural India for persons with schizophrenia, a range of stakeholders was engaged during consultation and during the feasibility and acceptability pilot [106]. For adolescents, there was limited stakeholder involvement reported in the design of services. However, there were studies in Chile and China for adolescent mental health that engaged stakeholders in the design [107,108].

#### 3.7.2. Identification of Intervention Beneficiaries

Recruitment of women with perinatal mental disorders via community resources (e.g., community health centers) was listed as a benefit in individual studies in China and India [68,109]. A trial in China recruited participants at the time of discharge from psychiatric facilities [51]. Informal community referral from organizations who had regular contact with vulnerable populations was done using local psychological idioms of distress in the Democratic Republic of Congo and Uganda [53,57,110]. For psychosis, there were no examples of case-finding components of the intervention. Instead participants were identified through inpatient or outpatient facilities [75,76,77,78,79,80,81,83,84,86,87] or through a previous epidemiological survey in China [82,85] and were already receiving treatment in all cases. For children and adolescents in settings of armed conflict, there were examples of whole classroom screening and referral by teachers in Burundi, Indonesia, Nepal, Sri Lanka, and South Sudan [93,94,98,100]. However, there were no data on improvement of access to needed mental health care reported by these studies. Some had a referral system built into the program for those identified as needing secondary mental health care [73,93,94,98,100].

#### 3.7.3. Recruitment

Where recruitment specifications for psychosis interventions were described, they included previous experience of delivering the psychosocial intervention [81,83], nursing diploma/degree [81] and, in the case of lay health workers, a minimum of ten years schooling and good interpersonal skills [77]. For child and adolescent interventions, interventions in schools included training of facilitators from amongst the school staff. For child and adolescent mental health promotion interventions, it was mostly class teachers in school and locally trained caregivers in the community [30]. For primary prevention interventions for children in LMICs, lay persons were recruited based on community residence, literacy, motivation, and ability to communicate with community members. Most of the studies used lay persons that did not have any previous training in health care. Across all the reviews, limited information was provided regarding training and supervision.

#### 3.7.4. Training and Acquiring Competency

Based on the information that was available for common mental disorders and perinatal disorders, trainings in psychological treatments were on average two-weeks in duration, with a range of a few days to two months, and supervision was provided typically weekly during the study period, with some studies having ad hoc or monthly supervision [26,46]. Training for psychosis interventions, mentioned in five studies [77,81,83,84,86], lasted between 12 hours [81] and six weeks [77]. Experts from high-income countries delivered trainings and supervision [53,56,57]. The selection, training and supervision of lay persons for child and adolescent interventions varied between studies with some trainings lasting up to a few months, but information on training was lacking for a number of the included interventions [44]. For some common mental disorders, when non-specialists were used for psychological care, the studies reported excluding some trainees because they did not achieve competence, which was evaluated with a written test or through clinical judgement from the trainers [26]. During the pilot of one study, the non-specialist providers tape-recorded sessions and a random selection of these were transcribed and subjected to content analysis [66]. Competency was assessed in two other studies, with no details reported [77,81].

#### 3.7.5. Assuring Quality

Supervision was present for those delivering some interventions. Limited information was provided in the reviews regarding if and how fidelity was monitored. In the Colombia prison study, asynchronous telepsychiatry allowed a non-specialist health worker to receive specialist supervision from a psychiatrist to assist treatment decisions for persons with depression [52]. Similarly, for perinatal care, only one study had a quality measure that comprised weekly review of written records as part of group supervision [66]. For services provided to persons with psychosis, in most studies, the lay community workers and non-mental health professionals received specialist input or supervision by psychiatrists or expert therapists [75,77,79,83,84,102]. In some cases, the lead delivery agent worked as part of multidisciplinary teams that included psychologists and psychiatric nurses [75,84]. For psychosis, a minority of studies measured process indicators, such as participant attendance at sessions [76,77] and psychiatrist contacts [77]. In one case, sessions were audio recorded to assess intervention fidelity [84]. In the evaluation of assertive community treatment in South Africa, fidelity to the international model of assertive community treatment was assessed using the Dartmouth Assertive Community Treatment Scale [75].

#### 3.7.6. Sustaining Motivation

There was limited information on formal evaluations to provide certification. Few trials reported compensation packages. Of those that did, examples included salaried community health workers [67], per diems during training [67], or reimbursement for relevant costs such as travel [67,104]. Some studies reported that participation was entirely volunteer-based [104].

#### 3.7.7. Delivery Formats

For perinatal care, most treatments were delivered at home in an individual format (n = 13). Six studies used a group format. Group formats were thought to activate social support for mothers [26,32]. For psychosis, several interventions involved group counselling or workshops for family members [78,82,85,87]. For alcohol use disorders, interventions were delivered in group formats and individually. For children and adolescents, interventions were predominantly in groups but also was to individuals, whole classrooms, mother-child dyads, teachers, pupils and parents in schools, and to caregivers in an orphanage. In humanitarian settings for children and adolescents, group interventions were predominantly delivered in schools, except for two studies that delivered community-based group programs for adolescents in Bosnia and Uganda [73,110].

#### 3.7.8. Integration into Other Platforms

In most cases, child and adolescent interventions appeared to be delivered in parallel to existing services, typically outpatient clinics. Integration into existing health systems—or potential facilitators or barriers to this—were not discussed [37,40]. Humanitarian settings typically led to delivery of psychological services alongside other nongovernmental programming such as protection programs and gender-based violence services in the Democratic Republic of Congo, Thailand, and Uganda [53,56,57,110]. Assertive community treatment teams for psychosis were typically a community-based extension that linked back into primary care and specialty psychiatric services, such as a program in South Africa [75]. For younger children and mothers, community mental health was integrated into nutrition and other maternal and child health programs. For example, the Lady Health Workers in Pakistan delivered the Thinking Healthy Program in the context of their standard perinatal maternal and child health programs [67]. For the prison study, mental health was integrated into the general primary care outreach services provided in the correctional facility [52].

#### 3.7.9. Implementation Barriers

Barriers related to both treatment delivery (e.g., poor adherence to treatment modality, economic cost of home visits and lack of private space) and the delivery agent (i.e., increased work pressure among existing health care workers and low motivation) [32]. For psychosis, potential barriers to intervention implementation included hypothesized stigmatization of mental health service use leading to low participation rates [80] and the cost of supervision for a lay health worker led model being prohibitively high [77]. The substantial resources needed to implement assertive community treatment and community-based rehabilitation were also highlighted [75,78]. Some authors discussed the modifications that had been made to these models of care, to make enhance feasibility in low resource settings. For example, the caseload in the trial of assertive community treatment was reduced and contacts were made less frequent [75].

### 3.8. Domain 6. Harms and Risks

One review highlighted the gap in reporting harms with non-specialist providers [49]. Home visits can be stigmatizing. For example, in India, some persons with schizophrenia and their family members in the Community care for People with Schizophrenia in India (COPSI) trial preferred to meet their mental health care providers in locations outside the home to avoid being stigmatized by neighbors for their participation in the program [111]. Another potential harm is that community components may be expensive to deliver; then, this potentially draws resources from other effective services [32,33].

## 4. Discussion

### 4.1. Community Components in Mental Health Care from Reviewed Literature

We identified community components reported in the academic literature based on a review-of-reviews strategy that included 23 systematic reviews of intervention trials in LMIC. Eight studies were identified relating to common mental disorders involving Brazil, China, Colombia, the Democratic Republic of Congo (DRC), Pakistan, Sri Lanka, Thailand, and Uganda. For perinatal mental disorders, 19 studies were identified involving Chile, China, India, Mexico, Pakistan, South Africa, Turkey, and Uganda. For psychosis, 13 studies were identified including China, India, Iran, South Africa, and Turkey. For substance use disorders, four studies involving from Mexico, Vietnam, and Malaysia. For mental disorders affecting children and adolescents, there were 122 studies, most of which were school-based programs, from LMIC across world regions.

**Figure 2:**
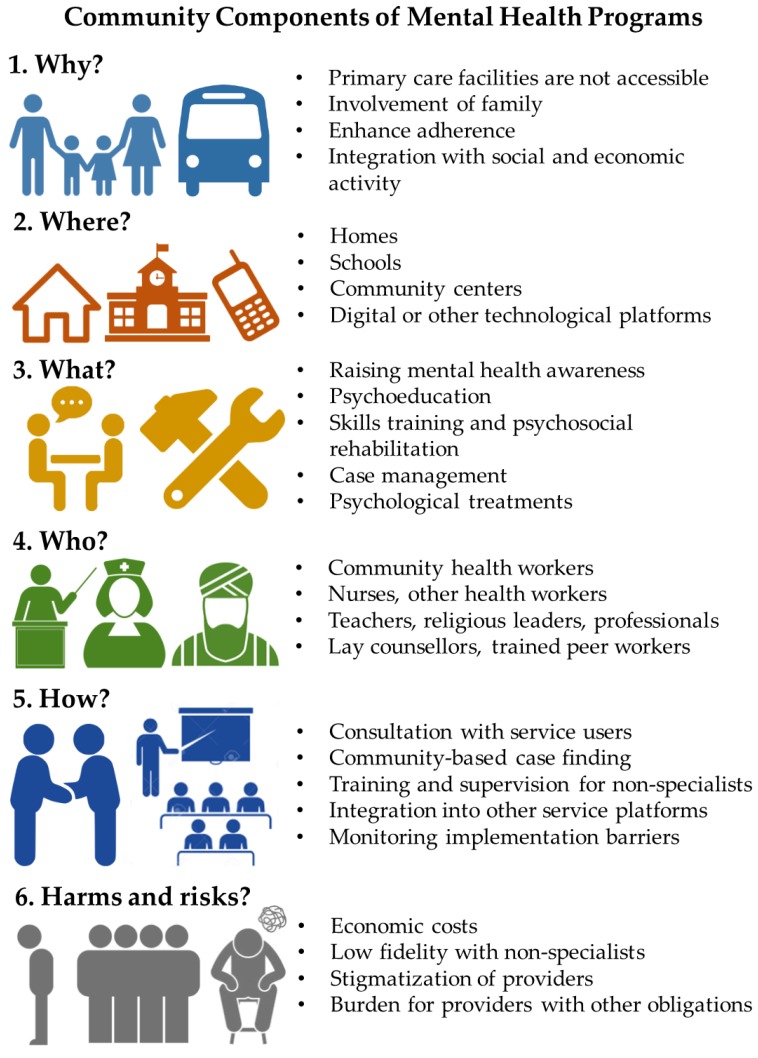
Domains for community mental health platforms.

Common reasons for using community platforms (Domain 1. Why?) were delivery of care when primary care services were not accessible or acceptable, enhancing quality of and engagement with clinical care, involving family members, and promoting social and economic inclusion (see Figure 2). The sites of community platforms (Domain 2. Where?) were homes, schools, other physical structures in the community, and technological platforms. One study highlighted the autonomy that comes with community designs, which allows for flexibility in organization and continuation of engagement after cessation of formal programs [104]. In a study of group interpersonal psychotherapy for caregivers of children with nodding syndrome in Uganda, the community platform (i.e., meeting under a tree in a rural area) was advantageous because the group continued to informally meet without a health worker present after the trial ended, and they started microfinance groups [112].

The common activities (Domain 3. What?) were population-wide awareness programs, psychoeducation, skills training, psychosocial rehabilitation, case management, and psychological treatments. In HIC settings, psychoeducation and crisis monitoring reduce involuntary hospitalizations [113], and there may be similar benefits in LMIC. One relevant study published after the included reviews, is VISHRAM, a grass-roots community-based mental health program in India. VISHRAM demonstrated a six-fold increase in contact coverage for depression by increasing mental health literacy in communities [114]. 

The facilitators (Domain 4. Who?) were community health workers, other health professionals, formal providers outside the health system, and non-formal providers. As a whole, these non-specialists were effective when delivering psychological treatments for adults [26] and other mental health services for children and adults [44,49]. Reflecting wider concerns in global health [115,116,117], three of the reviews called for more attention to low motivation among non-specialists, potential harms associated with non-specialist delivered care, and the potential burden to non-specialists, particularly among women [26,32,49]. The WPA has raised the issue of resistance among professionals and practitioners to community-oriented care [11,12], but this was not addressed in the reviews. Another area not addressed was how community-based approaches can facilitate greater service user autonomy in providing consent for mental health treatment [118]. One community stakeholder group not included in the current studies is law enforcement. Involvement of law enforcement is crucial to diverting persons with mental disorders from incarceration to treatment services, and mental health training for law enforcement personnel reduces human rights abuses of persons with mental disorders [119]. Police involvement is also recommended in the *Disease Control Priorities* [13]. A model for law enforcement and mental health collaboration through Crisis Intervention Teams (CIT) in Liberia has been piloted [120] and should be more rigorously evaluated as a key component of comprehensive community mental health care. Peers were also surprisingly absent from the currently evaluated models and research is needed to address this major gap.

The implementation processes (Domain 5. How?) involved consultation with mental health service users, community-based case detection, recruitment of facilitators, training and supervision of facilitators, assuring quality during implementation, sustaining motivation of facilitators, integration with other platforms, and addressing implementation barriers throughout the program. Integration with primary care varied widely.

Implementation approaches were characterized by manualized interventions but lacked structured guidelines and practices for evaluating competency, fidelity, and quality. Moreover, most reviews did not contain information on how quality would be monitored in the context of ongoing services after trial conditions concluded. Technological interventions were increasingly prominent in recent reviews. Technological innovations represent modalities for greater reach of services as well as greater quality of services when non-specialists have technological aids to guide their intervention [45], such as the avatar assisted delivery of the Thinking Healthy Program in Pakistan [121].

Potential negative consequences (Domain 6. Harms and Risks) included poor adherence to treatment in community settings, high economic costs of comprehensive community programs, maintaining motivation among non-specialists while competing with other professional, familial, and social demands, training and supervision to achieve minimum competency standards, and stigmatization of the community-based providers. There is also evidence that, in some contexts, community services may provide limited additional benefits over primary care services alone. In a recent cohort study in Nepal, persons with psychosis received community counseling and participated in peer support groups. These individuals did not have different outcomes when compared with a control condition only receiving primary care services and medication [122]. This may have been due to the benefit achieved with medication and underpowering to detect the additional benefit of community services. Moreover, the short duration of the trial may not have captured long-term benefits of the community component.

### 4.2. Agenda for Future Research on Community Components in Mental Health Care

Based on our findings, we have identified seven areas for future research on community mental health care.

#### 4.2.1. Recommendation 1. Develop Guidance on Standardized Reporting of Community Components for Mental Health Services

Measuring progress and improving practice is constricted because of the variability in what is reported about community mental health services. The WHO Mental Health Atlas includes some summary statistics to capture *what* is being done but has little to no information on *how* it is being done. For example, knowing that 49% of countries have involvement of service users and caregivers and that 55% of countries have anti-stigma programs [123] does not demonstrate the impact nor how this has been implemented. Standardized reporting would help to assess progress against the WPA guidance [11,12]. In Table 3, we recommend 12 domains for reporting of community components of mental health care as a starting point for documentation by organizations such as the WPA.

#### 4.2.2. Recommendation 2. Employ Implementation Science to Evaluate Community Components of Mental Health Care

The field of global mental health is at the appropriate stage to expand the use of theoretical perspectives and methodological approaches from implementation science. Not only is there a growing evidence base to support moving from effectiveness to implementation studies [26,28,49,124], but there has also been a concerted effort from the U.S. National Institute of Mental Health and Fogarty International Center, U.K. Medical Research Council, and Grand Challenges Canada to build capacity in implementation science research among LMIC and HIC global mental health researchers [125,126]. Through implementation science, studies can be designed and implemented to compare community components, delivery strategies, quality improvement approaches, and other issues that are essential to answer questions about how best to deliver community mental health care [124,127,128]. This should also include cost analysis and cost effectiveness studies to demonstrate the financial implications and benefits of community-based approaches.

#### 4.2.3. Recommendation 3. Study Approaches to Increase Service User and Family Involvement in Developing and Implementing Community Mental Health Services

The involvement of service users and their family members is a core component of current recommendations ranging from the WHO Mental Health Action Plan to the WPA guidance [8,11,12,13]. However, we found little discussion of the role of service users in our review. There has been limited research on service user involvement in LMIC, with a review reporting extremely limited participation of service users and caregivers in mental health system strengthening [129]. Therefore, approaches are needed to study how partnerships and collaborations with service users can best be developed and scaled up [129,130,131,132]. One approach has been training service users using a participatory photography research technique known as PhotoVoice and, following the training of service users, having them participate in training primary care and community health workers as well as engaging in theory of change workshops to design community and primary care services [133,134].

#### 4.2.4. Recommendation 4. Develop Tools to Study and Promote Competencies in Community Mental Health Care and use these for Research and Quality Improvement

There is increasing interest in identification of the competencies needed for delivery of effective mental health services in LMIC [135]. Moreover, a concern regarding community mental health services is the extent to which community service providers are competent enough to deliver the intended interventions [136]. Competencies have been identified for delivery of basic mental health services, tailored to primary care facilities [135]. Recently, several tools have been developed and tested to assess common factor competencies in psychological treatments with persons with mental illness [137,138,139]. Regarding community mental health care, current practices and guidelines suggest that there are at least six domains of activities that providers in community components may be required to do. These include establishing collaborations with service users and community organizations, promoting mental health literacy and improving attitudes, implementing mental health promotion programs, identifying persons with mental disorders and engaging them in care, delivering low intensity psychological treatments, and supporting psychosocial rehabilitation. Table 4 provides examples of competencies based on these six domains as well as examples of activities that would require those competencies. Our findings and the VISHRAM study mentioned above [114] underscore the importance of mental health literacy and the need for tools to easily evaluate community providers’ competency for increasing literacy. Scalable tools should be developed to evaluate these competencies; then, different strategies can be compared to determine how best to achieve and maintain these community mental health care competencies. This will help determine what cadres are most appropriate for service delivery across different contexts and assuring that minimum competency standards are achieved through training and supervision.

#### 4.2.5. Recommendation 5. Integrate and Evaluate Tools for Service Providers and Service Users to Enhance Reach and Effectiveness of Community Components

To achieve the objectives of community mental health care, it is crucial tools are developed to monitor practices and to improve effectiveness and sustainability of services. The Community Informant Detection Tool (CIDT) has been successfully used by community health volunteers to identify persons with mental disorder, using narrative vignettes, and then encourage them to seek care [140]. Measures also need to be developed to minimize potential harm of community-based services. These issues could be addressed through more supervision and by monitoring and reporting of adverse outcomes.

#### 4.2.6. Recommendation 6. Use Technology to Expand the Scope and Improve the Quality of Community Mental Health Services

How can increasing availability of self-help digital mobile applications be integrated into community components [29,45]? Some individuals using these self-help apps would benefit from additional human services and others may need to be referred for higher levels of care in the community or at health facilities. For example, the WHO’s *Self Help Plus* self-guided audio and book intervention is designed to be delivered in groups of 20–40, and although minimum facilitation is needed, the presence of a facilitator is helpful to assist participants with administering the audio selection and to refer persons for higher levels of care if needed [141]. This raises the question of how the apps can be utilized in a way that they are part of a continuum of care and not treatment cul-de-sacs that fail to connect persons with other care and supports. Naslund and colleagues [45] summarized innovations into five domains: supporting clinical care and educating health workers, mobile tools for facilitating diagnosis and detection of mental disorders, technologies for promoting treatment adherence and supporting recovery, online self-help program for individuals with mental disorders, and programs for substance misuse prevention and treatment. Future research should explore the impact of technology on other aspects of community mental health care.

#### 4.2.7. Recommendation 7. Better Integrate Community Platforms into Other Systems of Care

Effort is needed to integrate community platforms into existing government health systems and other care structures. A continuum of care should operate from light touch interventions into specialized services with community components along this pathway. This will facilitate scaling-up and scaling-out. van Ginneken and colleagues recently expanded the taxonomy to classify models of primary care integration and could be applied to these studies [142]. The taxonomy includes collaborative care models that utilize primary care and community care; collaborative care models that utilize community care only; the consultation-liaison model; the identification, referral, and sensitization model; the community outreach model; and the specialist-community model wherein primary care workers are training within specialist programs to provide community support.

## 5. Limitations

The review-of-reviews method allows for a broad scope but does not allow the in-depth examination that is possible when individual studies are reviewed in detail. Because of differences in extraction procedures across reviews, there was inconsistency in what information was available. Therefore, no meta-analysis was conducted. Because reviews were used, the most recent literature, which may address some of the key issues raised, was not included in the current study. Where appropriate, we have tried to include more recent studies in the discussion. Moreover, we did not capture pilot or feasibility studies unless they were included in the reviews, and these publications might have rich information on intervention design and rationale, fidelity measurement, and other issues. Our approach to community was a pragmatic and platform-based operationalization, but community mental health care can also be defined based on a philosophical perspective related to a population focus, socioeconomic context, preventive efforts, equitable access, team-based care, a life-course perspective, and cost-effectiveness for populations [12]. If a philosophical, rather than pragmatic, framing of community had been used, this would have affected which studies were included in our review.

## 6. Conclusions

Community components are vital to address global mental health needs and to rectify the stark gap between the burden of mental disorders and access to appropriate evidence-based interventions in LMIC. Community components in the current literature represent two important contributions to addressing the global burden of disease attributable to mental and substance use disorders. First, community components extend the reach of mental health services in settings where primary care and specialty services exist. The examples from settings such as Brazil, Chile, China, and South Africa demonstrate the extension of care. They create a continuum of care from the home setting through to primary care and then specialized psychiatric and psychological care. Second, community components are part of filling the gap in settings where mental health services do not exist in primary care. Examples from Burundi, Democratic Republic of Congo, northern Uganda, South Sudan, and the Thai–Myanmar border exemplify application of community mental health in conflict-affected settings. Future research will benefit from incorporating implementation science questions and methodology using, for example, hybrid designs to make the evaluation of effectiveness and implementation outcomes core to any studies exploring community components in LMIC. We also recommend standardizing reporting for community components and for increased attention to evaluating competencies. There are promising developments for integration of new instruments and technologies utilized in community components, but we caution against stand-alone technology that does not link with other community and health system components. Going forward, it will be vital to involve more service users in design and implementation and to better document this process for community mental health services.

## Figures and Tables

**Table 1:** Characteristics of included reviews.

Author, Year	Categories of Mental Disorders
Asher et al., 2017 [28]	Psychoses
Arjadi et al., 2015 [29]	Common mental disorders
Barry et al., 2013 [30]	Child and adolescent disorders
Chibanda et al., 2015 [31]	Common mental disorders
Chowdhary et al., 2014 [32]	Perinatal mental disorders
Chowdhary et al., 2014 [33]	Common mental disorders, Perinatal mental disorders
Clarke et al., 2013 [34]	Perinatal mental disorders
Cuijpers et al., 2017 [35]	Common mental disorders, Perinatal mental disorders
De Silva et al., 2013 [36]	Common mental disorders, Psychoses
Fazel et al., 2014 [37]	Child and adolescent disorders
Iemmi et al., 2016 [38]	Psychoses
Jordans et al., 2009 [39]	Child and adolescent disorders
Jordans et al., 2016 [40]	Child and adolescent disorders
Kieling et al., 2011 [41]	Child and adolescent disorders
Klasen et al., 2013 [42]	Child and adolescent disorders
Lund et al., 2011 [43]	Common mental disorders
Mutamba et al., 2013 [44]	Common mental disorders, Child and adolescent disorders
Naslund et al., 2017 [45]	Common mental disorders, Psychoses
Rahman et al., 2013 [46]	Perinatal mental disorders
Rane et al., 2017 [47]	Substance use disorders
Singla et al., 2017 [26]	Common mental disorders, Perinatal mental disorders
Tyrer et al., 2014 [48]	Child and adolescent disorders
van Ginneken et al., 2013 [49]	Common mental disorders, Perinatal mental disorders, Psychoses, Substance use disorders, Child and adolescent disorders

**Table 2:** AMSTAR 2 Quality Assessment of Systematic Reviews.

	(1) Question and Inclusion	(2) Protocol	(3) Study Design	(4) Comprehensive Search	(5) Study Selection	(6) Data Extraction	(7) Excluded Studies Justification	(8) Included Studies Details	(9) Risk of Bias (RoB)	(10) Funding Sources	(11) Statistical Methods	(12) RoB on meta-analysis	(13) RoB in individual Studies	(14) Explanation for Heterogeneity	(15) Publication Bias	(16) Conflict of Interest
Asher et al., 2017 [28]	Yes	Yes	Yes	Yes	Yes	No	Yes	Yes	Yes	No	Yes	Yes	Yes	Yes	Yes	Yes
Arjadi et al., 2015 [29]	Yes	Yes	Yes	Yes	No	No	No	Yes	Partial Yes	No	N/A	N/A	Yes	Yes	Yes	Yes
Barry et al., 2013 [30]	Yes	Yes	Yes	Yes	Yes	Yes	No	Yes	Yes	No	N/A	N/A	Yes	Yes	Yes	Yes
Chibanda et al., 2015 [31]	Yes	Yes	Yes	Partial Yes	Yes	Yes	Yes	Yes	Yes	No	N/A	N/A	Yes	Yes	Yes	Yes
Chowdhary et al., 2014 [32]	Yes	Yes	Yes	Yes	Yes	Yes	No	Yes	Yes	No	N/A	N/A	Yes	Yes	Yes	Yes
Chowdhary et al., 2014 [33]	Yes	Yes	Yes	Yes	Yes	Yes	No	Yes	Yes	No	Yes	Yes	Yes	Yes	Yes	Yes
Clarke et al., 2013 [34]	Yes	Yes	Yes	Yes	Yes	Yes	No	Yes	Yes	No	Yes	Yes	Yes	Yes	Yes	Yes
Cuijpers et al., 2017 [35]	Yes	Yes	Yes	Partial Yes	Yes	Yes	No	Yes	Yes	No	Yes	Yes	Yes	Yes	Yes	Yes
De Silva et al., 2013 [36]	Yes	Yes	Yes	Yes	Yes	Yes	Partial Yes	Yes	Yes	No	Yes	Yes	Yes	Yes	Yes	Yes
Fazel et al., 2014 [37]	Yes	Partial Yes	Yes	Partial Yes	Yes	Yes	No	Yes	Partial Yes	No	N/A	N/A	No	Yes	Yes	Yes
Iemmi et al., 2016 [38]	Yes	Yes	Yes	Yes	Yes	Yes	Yes	Yes	Yes	No	Yes	Yes	Yes	Yes	Yes	Yes
Jordans et al., 2009 [39]	Yes	Yes	Yes	Yes	Yes	Yes	No	Yes	Partial Yes	No	N/A	N/A	Yes	Yes	Yes	Yes
Jordans et al., 2016 [40]	Yes	Yes	Yes	Yes	Yes	Yes	Yes	Yes	Yes	No	N/A	N/A	Yes	Yes	Yes	Yes
Kieling et al., 2011 [41]	Yes	Partial Yes	No	Partial Yes	No	No	No	Partial Yes	No	No	N/A	N/A	No	Yes	No	Yes
Klasen et al., 2013 [42]	Yes	Yes	Yes	Yes	No	No	No	Yes	Yes	No	N/A	N/A	No	Yes	Yes	Yes
Lund et al., 2011 [43]	Yes	Yes	Yes	Yes	Yes	Yes	No	Yes	Partial Yes	No	N/A	N/A	Yes	Yes	Yes	Yes
Mutamba et al., 2013 [44]	Yes	Yes	Yes	Partial Yes	Yes	Yes	No	Yes	Yes	No	Yes	Yes	Yes	Yes	Yes	Yes
Naslund et al., 2017 [45]	Yes	Yes	Yes	Yes	Yes	Yes	No	Yes	Partial Yes	No	N/A	N/A	No	Yes	Yes	Yes
Rahman et al., 2013 [46]	Yes	Yes	Yes	Yes	Yes	Yes	No	Yes	Yes	No	Yes	Yes	Yes	Yes	Yes	Yes
Rane et al., 2017 [47]	Yes	Yes	Yes	Partial Yes	Yes	Yes	No	Yes	Partial Yes	No	N/A	N/A	No	Yes	Yes	Yes
Singla et al., 2017 [26]	Yes	Yes	Yes	Yes	Yes	Yes	Yes	Yes	Yes	No	Yes	Yes	No	Yes	Yes	Yes
Tyrer et al., 2014 [48]	Yes	Yes	Yes	Yes	Yes	Yes	No	Yes	Yes	No	N/A	N/A	Yes	Yes	Yes	Yes
van Ginneken et al., 2013 [49]	Yes	Yes	Yes	Yes	Yes	Yes	Yes	Yes	Yes	Yes	Yes	Yes	Yes	Yes	Yes	Yes
Total, *N* (%) *	23 (100%)	21 (91%)	22 (96%)	17 (74%)	20 (87%)	19 (83%)	6 (26%)	22 (96%)	16 (70%)	1 (4%)	10 (100%)	10 (100%)	17 (74%)	23 (100%)	22 (96%)	23 (100%)

Abbreviations: RoB, Risk of Bias. * Percent is based on number of eligible reviews per domain.

**Table 3:** Guidance for reporting of community mental health components.

Domain	Information
*Involvement of service users and family members*	How are service users and family members engaged in selection, design, implementation, and evaluation of community components?
*2*. *Involvement of other stakeholders*	In addition to service users and caregivers, how were other stakeholders in the community engaged in the design, implementation, and evaluation? This may include potential cadres responsible for delivery and supervision of the program.
*3*. *Rationale for use of community components*	Why was a community approach selected, and what specific community component was chosen? Include formative research, literature reviews, theory of change workshops and other approaches employed; report the evidence base (e.g., GRADE scoring) for selected approach when available.
*4*. *Procedures to assure equity, promotion of human rights, and protection from stigma and discrimination*	How do services equitably account for gender, ethnicity, socioeconomic status, and other social factors? What mechanisms are in place to monitor and promote human rights, e.g., *QualityRights*; (understanding informed consent before patients decide about treatment without feeling coerced)? How are stigma and discrimination monitored and addressed?
*5*. *Scope of activities to address mental health literacy, and prevention/promotion*	What activities are included in the community component to address the multiple tiers of comprehensive services, including how is mental health literacy increased? What is done to address universal, targeted, or indicated prevention?
*6*. *Treatment and rehabilitation services*	What treatments are included in the community component; and how are livelihood and quality of life addressed with psychosocial rehabilitation services?
*7*. *Platforms for service delivery*	Where are the platforms for the community component; how was it selected and what are the facilitators and barriers?
*8*. *Cadres and competencies of community-based service providers*	Who is delivering the intervention; how were they selected, trained, and supervised; how is competency evaluated and promoted; how is the mental health and quality of life of service providers monitored?
*9*. *Integration into existing healthcare systems*	How is the community program integrated into existing healthcare system; what are referral processes in stepped-care approaches?
*10*. *Implementation procedures for establishing, sustaining, evolving, and scaling-up services*	How was the intervention adapted for the specific context; how are fidelity and quality monitored; how is the intervention adapted over time to adjust to community needs and resources; how much do the activities cost; what are the policies, manuals, and material resources needed for initiation, sustaining, and scaling up the community component?
*11*. *Technologies used for community components*	What technologies are used for delivery, monitoring fidelity and quality, promoting adherence, etc. (e.g., person-to-person contact through phone; apps on mobile devices; internet-based services)?
*12*. *Adverse events and unintended outcomes*	What adverse events were experienced by participants; did community providers experience adverse outcomes; were there unintended consequences?

**Table 4:** Competencies needed for community mental health care.

Domains	Competencies	Examples
*A*. *Partnerships and collaboration with service users, families, and other organizations*	Engaging with service users and family members Empowering services users for participation in community components Engaging with other service sectors: physical health, education, livelihood, law enforcement, and social programs	Community based participatory techniques (e.g., rural appraisal, participatory policy analysis, theory of change workshops with service users, PhotoVoice with service users) Integration of maternal and child mental health into nutrition and reproductive health services Integration of stress reduction and substance use risk reduction into the workplace Integration of conflict reduction programs and peace programs into schools and communities Training Crisis Intervention Teams (CIT)
*B*. *Mental health literacy and attitudes*	Teaching basic mental health literacy Reducing stigma against persons with mental illness Psychoeducation for specific conditions Respecting the rights of persons with mental illness Awareness and reporting of human rights abuses Promoting social inclusion Awareness of co-occurring and chronic illnesses	Conducting individual, family, and community psychoeducation and mental health literacy programs (e.g., VISHRAM in India) Designing radio program, street dramas, etc. Training for inclusion based on United Nations Convention on the Rights of Persons with Disabilities for service users, service providers, and legal and law enforcement communities Training on treatment of chronic illnesses based on the WHO Innovative Care for Chronic Conditions: Building Blocks for Action Designing and implementing social contact interventions
*C*. *Mental health promotion and mental illness prevention*	Promoting hope, coping behaviors, and self-care Training adolescents and adults on life skills Delivering parenting programs Promoting community policies and legislation for risk reduction	Manualized interventions such as Life-training Skills, Good Behavior Game, and Classroom Based Intervention Training caregivers about child development Enforcing tax on alcohol and restricting access to firearms and pesticides Addressing structural violence (exclusion) and direct violence
*D*. *Identification of and service engagement for persons with mental illness*	Ability to perform pro-active case finding, and/or universal or targeted screening Facilitating treatment initiation and referrals to assure entry into care	Community Informant Detection Tools (CIDT) for pro-active case finding Using and interpreting validated screening tools Using technology to facilitate referrals and monitor entry into care
*E*. *Treatment of persons with mental illness*	Promoting equitable access to services Treatment competencies for psychological therapies, and medication adherence Ability to adjust treatment plans for personalized care	Low intensity psychological treatments: Thinking Healthy Program, Problem Management Plus, Interpersonal Psychotherapy for Groups, Healthy Activity Program, Counseling for Alcohol Problems, Friendship Bench
*F*. *Psychosocial rehabilitation and livelihood promotion*	Training on employment readiness skills Using recovery-based engagement models Promoting self-management	Community Based Rehabilitation Occupational therapy programs Engagement of family in supporting recovery Referral to microfinance, microcredit programs who support participation of persons with mental illness and family members

## References

[1] Allen J., Balfour R., Bell R., Marmot M. (2014). Social determinants of mental health. Int. Rev. Psychiatry.

[2] Druss B.G., von Esenwein S.A., Compton M.T., Rask K.J., Zhao L., Parker R.M. (2010). A Randomized Trial of Medical Care Management for Community Mental Health Settings: The Primary Care Access, Referral, and Evaluation (PCARE) Study. Am. J. Psychiatry.

[3] Marmot M., Friel S., Bell R., Houweling T.A.J., Taylor S. (2008). Closing the gap in a generation: Health equity through action on the social determinants of health. Lancet.

[4] Goldberg D., Huxley P. (2012). Mental Illness in the Community: The Pathway to Psychiatric Care.

[5] Evans-Lacko S., Corker E., Williams P., Henderson C., Thornicroft G. (2014). Effect of the Time to Change anti-stigma campaign on trends in mental-illness-related public stigma among the English population in 2003–13: An analysis of survey data. Lancet Psychiatry.

[6] Jorm A.F. (2012). Mental health literacy: Empowering the community to take action for better mental health. Am. Psychol..

[7] Brekke J., Kay D.D., Lee K.S., Green M.F. (2005). Biosocial pathways to functional outcome in schizophrenia. Schizophr. Res..

[8] WHO (2013). Mental Health Action Plan 2013–2020.

[9] World Health Organization (2012). WHO QualityRights Tool Kit.

[10] United Nations (2006). Convention on the Rights of Persons with Disabilities.

[11] Thornicroft G., Alem A., Antunes Dos Santos R., Barley E., Drake R.E., Gregorio G., Hanlon C., Ito H., Latimer E., Law A. (2010). WPA guidance on steps, obstacles and mistakes to avoid in the implementation of community mental health care. World Psychiatry.

[12] Thornicroft G., Deb T., Henderson C. (2016). Community mental health care worldwide: Current status and further developments. World Psychiatry.

[13] Patel V., Chisholm D., Parikh R., Charlson F.J., Degenhardt L., Dua T., Ferrari A.J., Hyman S., Laxminarayan R., Levin C. (2016). Addressing the burden of mental, neurological, and substance use disorders: Key messages from Disease Control Priorities, 3rd edition. Lancet.

[14] Thornicroft G., Chatterji S., Evans-Lacko S., Gruber M., Sampson N., Aguilar-Gaxiola S., Al-Hamzawi A., Alonso J., Andrade L., Borges G. (2017). Undertreatment of people with major depressive disorder in 21 countries. Br.J. Psychiatry.

[15] Thirthalli J., Channaveerachari N., Subbakrishna D., Cottler L., Varghese M., Gangadhar B. (2011). Prospective study of duration of untreated psychosis and outcome of never-treated patients with schizophrenia in India. Indian J. Psychiatry.

[16] Drew N., Funk M., Tang S., Lamichhane J., Chávez E., Katontoka S. (2011). Human rights violations of people with mental and psychosocial disabilities: An unresolved global crisis. Lancet.

[17] Asher L., Fekadu A., Teferra S., De Silva M., Pathare S., Hanlon C. (2017). “I cry every day and night, I have my son tied in chains”: Physical restraint of people with schizophrenia in community settings in Ethiopia. Glob. Health.

[18] Ofori-Atta A., Attafuah J., Jack H., Baning F., Rosenheck R. (2018). Joining psychiatric care and faith healing in a prayer camp in Ghana: Randomised trial. Br.J. Psychiatry.

[19] Patel V., Burns J.K., Dhingra M., Tarver L., Kohrt B.A., Lund C. (2018). Income inequality and depression: A systematic review and meta-analysis of the association and a scoping review of mechanisms. World Psychiatry.

[20] Lund C., Stansfeld S., De Silva M., Patel V., Minas H., Cohen A., Prince M.J. (2014). Social determinants of mental health. Global Mental Health: Principles and Practice.

[21] Semrau M., Evans-Lacko S., Koschorke M., Ashenafi L., Thornicroft G. (2015). Stigma and discrimination related to mental illness in low- and middle-income countries. Epidemiol. Psychiatr. Sci..

[22] Hanlon C., Luitel N.P., Kathree T., Murhar V., Shrivasta S., Medhin G., Ssebunnya J., Fekadu A., Shidhaye R., Petersen I. (2014). Challenges and opportunities for implementing integrated mental health care: A district level situation analysis from five low- and middle-income countries. PLoS ONE.

[23] Gwaikolo W.S., Kohrt B.A., Cooper J.L. (2017). Health system preparedness for integration of mental health services in rural Liberia. BMC Health Serv. Res..

[24] Angdembe M., Kohrt B.A., Jordans M., Rimal D., Luitel N.P. (2017). Situational analysis to inform development of primary care and community-based mental health services for severe mental disorders in Nepal. Int. J. Ment. Health Syst..

[25] The World Bank Word Bank Country and Lending Groups. https://datahelpdesk.worldbank.org/knowledgebase/articles/906519-world-bank-country-and-lending-groups.

[26] Singla D.R., Kohrt B.A., Murray L.K., Anand A., Chorpita B.F., Patel V. (2017). Psychological treatments for the world: Lessons from low- and middle-income countries. Annu. Rev. Clin. Psychol..

[27] Shea B.J., Reeves B.C., Wells G., Thuku M., Hamel C., Moran J., Moher D., Tugwell P., Welch V., Kristjansson E. (2017). AMSTAR 2: A critical appraisal tool for systematic reviews that include randomised or non-randomised studies of healthcare interventions, or both. BMJ.

[28] Asher L., Patel V., De Silva M.J. (2017). Community-based psychosocial interventions for people with schizophrenia in low and middle-income countries: Systematic review and meta-analysis. BMC Psychiatry.

[29] Arjadi R., Nauta M.H., Chowdhary N., Bockting C.L.H. (2015). A systematic review of online interventions for mental health in low and middle income countries: A neglected field. Glob. Ment. Health.

[30] Barry M.M., Clarke A.M., Jenkins R., Patel V. (2013). A systematic review of the effectiveness of mental health promotion interventions for young people in low and middle income countries. BMC Public Health.

[31] Chibanda D., Cowan F.M., Healy J.L., Abas M., Lund C. (2015). Psychological interventions for Common Mental Disorders for People Living With HIV in Low- and Middle-Income Countries: Systematic review. Trop. Med. Int. Health.

[32] Chowdhary N., Sikander S., Atif N., Singh N., Ahmad I., Fuhr D.C., Rahman A., Patel V. (2014). The content and delivery of psychological interventions for perinatal depression by non-specialist health workers in low and middle income countries: A systematic review. Best Pract. Res. Clin. Obstet. Gynaecol..

[33] Chowdhary N., Jotheeswaran A., Nadkarni A., Hollon S., King M., Jordans M., Rahman A., Verdeli H., Araya R., Patel V. (2014). The methods and outcomes of cultural adaptations of psychological treatments for depressive disorders: A systematic review. Psychol. Med..

[34] Clarke K., King M., Prost A. (2013). Psychosocial Interventions for Perinatal Common Mental Disorders Delivered by Providers Who Are Not Mental Health Specialists in Low- and Middle-Income Countries: A Systematic Review and Meta-Analysis. PLoS Med..

[35] Cuijpers P., Karyotaki E., Reijnders M., Purgato M., Barbui C. (2018). Psychotherapies for depression in low- and middle-income countries: A meta-analysis. World Psychiatry.

[36] De Silva M.J., Cooper S., Li H.L., Lund C., Patel V. (2013). Effect of psychosocial interventions on social functioning in depression and schizophrenia: Meta-analysis. Br. J. Psychiatry.

[37] Fazel M., Patel V., Thomas S., Tol W. (2014). Mental health interventions in schools in low-income and middle-income countries. Lancet Psychiatry.

[38] Iemmi V., Blanchet K., Gibson L.J., Kumar K.S., Rath S., Hartley S., Murthy G.V.S., Patel V., Weber J., Kuper H. (2016). Community-based rehabilitation for people with physical and mental disabilities in low- and middle-income countries: A systematic review and meta-analysis. J. Dev. Eff..

[39] Jordans M.J.D., Tol W.A., Komproe I.H., De Jong J.T.V.M. (2009). Systematic review of evidence and treatment approaches: Psychosocial and mental health care for children in war. Child Adolesc. Ment. Health.

[40] Jordans M.J., Pigott H., Tol W.A. (2016). Interventions for Children Affected by Armed Conflict: A Systematic Review of Mental Health and Psychosocial Support in Low- and Middle-Income Countries. Curr. Psychiatry Rep..

[41] Kieling C., Baker-Henningham H., Belfer M., Conti G., Ertem I., Omigbodun O., Rohde L.A., Srinath S., Ulkuer N., Rahman A. (2011). Child and adolescent mental health worldwide: Evidence for action. Lancet.

[42] Klasen H., Crombag A.C. (2013). What works where? A systematic review of child and adolescent mental health interventions for low and middle income countries. Soc. Psychiatry Psychiatr. Epidemiol..

[43] Lund C., De Silva M., Plagerson S., Cooper S., Chisholm D., Das J., Knapp M., Patel V. (2011). Poverty and mental disorders: Breaking the cycle in low-income and middle-income countries. Lancet.

[44] Mutamba B.B., van Ginneken N., Smith Paintain L., Wandiembe S., Schellenberg D. (2013). Roles and effectiveness of lay community health workers in the prevention of mental, neurological and substance use disorders in low and middle income countries: A systematic review. BMC Health Serv. Res..

[45] Naslund J.A., Aschbrenner K.A., Araya R., Marsch L.A., Unützer J., Patel V., Bartels S.J. (2017). Digital technology for treating and preventing mental disorders in low-income and middle-income countries: A narrative review of the literature. Lancet Psychiatry.

[46] Rahman A., Fisher J., Bower P., Luchters S., Thach T., Yasamy M.T., Saxena S., Waheed W. (2013). Interventions for common perinatal mental disorders in women in low- and middle-income countries: A systematic review and meta-analysis. Bull. World Health Org..

[47] Rane A., Church S., Bhatia U., Orford J., Velleman R., Nadkarni A. (2017). Psychosocial interventions for addiction-affected families in Low and Middle Income Countries: A systematic review. Addict. Behav..

[48] Tyrer R.A., Fazel M. (2014). School and Community-Based Interventions for Refugee and Asylum Seeking Children: A Systematic Review. PLoS ONE.

[49] van Ginneken N., Tharyan P., Lewin S., Rao G.N., Meera S.M., Pian J., Chandrashekar S., Patel V. (2013). Non-specialist health worker interventions for the care of mental, neurological and substance-abuse disorders in low- and middle-income countries. Cochrane Database Syst. Rev..

[50] Hungerbuehler I., Valiengo L., Loch A.A., Rössler W., Gattaz W.F. (2016). Home-Based Psychiatric Outpatient Care Through Videoconferencing for Depression: A Randomized Controlled Follow-Up Trial. JMIR Ment Health.

[51] Hu X., Wang Y. (2007). Yi yu zheng zong he shi jia ting zhi liao: 76 li dan mang sui ji dui zhao (Synthetical family treatment for depression: A randomized-controlled single-blind study among 76 cases). J. Clin. Rehabil. Tissue Eng. Res..

[52] Barrera-Valencia C., Benito-Devia A.V., Vélez-Álvarez C., Figueroa-Barrera M., Franco-Idárraga S.M. (2017). Cost-effectiveness of synchronous vs. asynchronous telepsychiatry in prison inmates with depression. Rev. Colomb. Psiquiatr. (Engl. Ed.).

[53] Bass J.K., Annan J., McIvor Murray S., Kaysen D., Griffiths S., Cetinoglu T., Wachter K., Murray L.K., Bolton P.A. (2013). Controlled Trial of Psychotherapy for Congolese Survivors of Sexual Violence. New Engl. J. Med..

[54] Ali B.S., Rahbar M.H., Naeem S., Gul A., Mubeen S., Iqbal A. (2003). The effectiveness of counseling on anxiety and depression by minimally trained counselors: A randomized controlled trial. Am. J. Psychoth..

[55] Marasinghe R.B., Edirippulige S., Kavanagh D., Smith A., Jiffry M.T.M. (2012). Effect of mobile phone-based psychotherapy in suicide prevention: A randomized controlled trial in Sri Lanka. J. Telemed. Telecare.

[56] Bolton P., Lee C., Haroz E.E., Murray L., Dorsey S., Robinson C., Ugueto A.M., Bass J. (2014). A Transdiagnostic Community-Based Mental Health Treatment for Comorbid Disorders: Development and Outcomes of a Randomized Controlled Trial among Burmese Refugees in Thailand. PLoS Med..

[57] Bolton P., Bass J., Neugebauer R., Verdeli H., Clougherty K.F., Wickramaratne P., Speelman L., Ndogoni L., Weissman M. (2003). Group interpersonal psychotherapy for depression in rural Uganda: A randomized controlled trial. JAMA.

[58] Tiwari A., Fong D.Y.T., Yuen K.H., Yuk H., Pang P., Humphreys J., Bullock L. (2010). Effect of an Advocacy Intervention on Mental Health in Chinese Women Survivors of Intimate Partner Violence A Randomized Controlled Trial. JAMA.

[59] Gao L.-L., Luo S.-Y., Chan S.W.-C. (2012). Interpersonal psychotherapy-oriented program for Chinese pregnant women: Delivery, content, and personal impact. Nurs. Health Sci..

[60] Gao L.-l., Chan S.W.-c., Li X., Chen S., Hao Y. (2010). Evaluation of an interpersonal-psychotherapy-oriented childbirth education programme for Chinese first-time childbearing women: A randomised controlled trial. Int. J. Nurs. Stud..

[61] Jiang L., Wang Z.-Z., Qiu L.-R., Wan G.-B., Lin Y., Wei Z. (2014). Psychological intervention for postpartum depression. J. Huazhong Univ. Sci. Technol. [Med. Sci.].

[62] Ngai F.W., Wong P.W.C., Leung K.Y., Chau P.H., Chung K.F. (2015). The Effect of Telephone-Based Cognitive-Behavioral Therapy on Postnatal Depression: A Randomized Controlled Trial. Psychother. Psychosom..

[63] Petersen I., Hancock J.H., Bhana A., Govender K. (2014). A group-based counselling intervention for depression comorbid with HIV/AIDS using a task shifting approach in South Africa: A randomized controlled pilot study. J. Affect. Disord..

[64] Le Roux I.M., Tomlinson M., Harwood J.M., O’Connor M.J., Worthman C.M., Mbewu N., Stewart J., Hartley M., Swendeman D., Comulada W.S. (2013). Outcomes of home visits for pregnant mothers and their infants: A cluster randomised controlled trial. AIDS (Lond. Engl.).

[65] Cooper P.J., Landman M., Tomlinson M., Molteno C., Swartz L., Murray L. (2002). Impact of a mother—Infant intervention in an indigent peri-urban South African context. Br. J. Psychiatry.

[66] Cooper P.J., Tomlinson M., Swartz L., Landman M., Molteno C., Stein A., McPherson K., Murray L. (2009). Improving quality of mother-infant relationship and infant attachment in socioeconomically deprived community in South Africa: Randomised controlled trial. Br. Med. J..

[67] Rahman A., Malik A., Sikander S., Roberts C., Creed F. (2008). Cognitive behaviour therapy-based intervention by community health workers for mothers with depression and their infants in rural Pakistan: A cluster-randomised controlled trial. Lancet.

[68] Hughes M. (2009). Randomised, Controlled Trial of a Perinatal Psycho-Social Intervention for Postnatal Depression in Goa, India.

[69] Hirani S.S., Karmaliani R., McFarlane J., Asad N., Madhani F., Shehzad S., Ali N.A. (2010). Development of an economic skill building intervention to promote women's safety and child development in Karachi, Pakistan. Issues Ment. Health Nurs..

[70] Aracena M., Krause M., Pérez C., Méndez M.J., Salvatierra L., Soto M., Pantoja T., Navarro S., Salinas A., Farah C. (2009). A cost-effectiveness evaluation of a home visit program for adolescent mothers. J. Health Psychol..

[71] Lara M.A., Navarro C., Navarrete L. (2010). Outcome results of a psycho-educational intervention in pregnancy to prevent PPD: A randomized control trial. J. Affect. Disord..

[72] Langer A., Farnot U., Garcia C., Barros F., Victora C., Belizan J.M., Villar J. (1996). The Latin American trial of psychosocial support during pregnancy: Effects on mother's wellbeing and satisfaction. Soc. Sci. Med..

[73] Dybdahl R. (2001). Children and mothers in war: An outcome study of a psychosocial intervention program. Child Dev..

[74] Tezel A., Gözüm S. (2006). Comparison of effects of nursing care to problem solving training on levels of depressive symptoms in post partum women. Patient Educa. Couns..

[75] Botha U.A., Koen L., Joska J.A., Hering L.M., Oosthuizen P.P. (2010). Assessing the efficacy of a modified assertive community-based treatment programme in a developing country. BMC Psychiatry.

[76] Cai J., Zhu Y., Zhang W., Wang Y., Zhang C. (2015). Comprehensive family therapy: An effective approach for cognitive rehabilitation in schizophrenia. Neuropsychiatr. Dis. Treat..

[77] Chatterjee S., Naik S., John S., Dabholkar H., Balaji M., Koschorke M., Varghese M., Thara R., Weiss H.A., Williams P. (2014). Effectiveness of a community-based intervention for people with schizophrenia and their caregivers in India (COPSI): A randomised controlled trial. Lancet.

[78] Chatterjee S., Patel V., Chatterjee A., Weiss H.A. (2003). Evaluation of a community-based rehabilitation model for chronic schizophrenia in rural India. Br.J. Psychiatry J. Ment. Sci..

[79] Ghadiri Vasfi M., Moradi-Lakeh M., Esmaeili N., Soleimani N., Hajebi A. (2015). Efficacy of aftercare services for people with severe mental disorders in Iran: A randomized controlled trial. Psychiatr. Serv..

[80] Hegde S., Rao S.L., Raguram A., Gangadhar B.N. (2012). Addition of home-based cognitive retraining to treatment as usual in first episode schizophrenia patients: A randomized controlled study. Indian J. Psychiatry.

[81] Li Z., Arthur D. (2005). Family education for people with schizophrenia in Beijing, China: Randomised controlled trial. Br.J. Psychiatry.

[82] Ran M.-S., Xiang M.-Z., Chan C.L.-W., Leff J., Simpson P., Huang M.-S., Shan Y.-H., Li S.-G. (2003). Effectiveness of psychoeducational intervention for rural Chinese families experiencing schizophrenia. A randomised controlled trial. Soc. Psychiatry Psychiatr. Epidemiol..

[83] Sharifi V., Tehranidoost M., Yunesian M., Amini H., Mohammadi M., Jalali Roudsari M. (2012). Effectiveness of a low-intensity home-based aftercare for patients with severe mental disorders: A 12-month randomized controlled study. Commun. Ment. Health J..

[84] Sungur M.B., Soygur H., Guner P., Ustun B., Cetin I., Falloon I.R. (2011). Identifying an optimal treatment for schizophrenia: A 2-year randomized controlled trial comparing integrated care to a high-quality routine treatment. Int. J. Psychiatry Clin. Pract..

[85] Xiang M., Ran M., Li S. (1994). A controlled evaluation of psychoeducational family intervention in a rural Chinese community. Br. J. Psychiatry.

[86] Xiong W., Phillips M.R., Hu X., Wang R., Dai Q., Kleinman J., Kleinman A. (1994). Family-based intervention for schizophrenic patients in China: A randomised controlled trial. Br.J. Psychiatry.

[87] Zhang M., Wang M., Li J., Phillips M.R. (1994). Randomised-control trial of family intervention for 78 first-episode male schizophrenic patients: An 18-month study in Suzhou, Jiangsu. Br. J. Psychiatry.

[88] Cruz Almanza M.D.L.A., Gaona Márquez L., Sánchez Sosa J.J. (2006). Empowering women abused by their problem drinker spouses: Effects of a cognitive-behavioral intervention. Salud Ment..

[89] Tiburcio M., Natera G. (2003). Evaluación de un modelo de intervención breve para familiares de usuarios de alcohol y drogas. Un estudio piloto. Salud Ment..

[90] Li L., Hien N.T., Lin C., Tuan N.A., Tuan L.A., Farmer S.C., Detels R. (2014). An intervention to improve mental health and family well-being of injecting drug users and family members in Vietnam. Psychol. Addict. Behav..

[91] Baharudin D.F., Mohd Hussin A.H., Sumari M., Mohamed S., Zakaria M.Z., Sawai R.P. (2014). Family intervention for the treatment and rehabilitation of drug addiction: An exploratory study. J. Subst. Use.

[92] Jordans M.J., Tol W.A., Komproe I.H., Susanty D., Vallipuram A., Ntamatumba P., Lasuba A.C., de Jong J.T. (2010). Development of a multi-layered psychosocial care system for children in areas of political violence. Int. J. Ment. Health Syst..

[93] Jordans M.J.D., Komproe I.H., Tol W.A., Kohrt B.A., Luitel N.P., Macy R.D., de Jong J.T.V.M. (2010). Evaluation of a classroom-based psychosocial intervention in conflict-affected Nepal: A cluster randomized controlled trial. J. Child Psychol. Psychiatry Allied Discip..

[94] Tol W., Komproe I., Jordans M., Ndayisaba A., Ntamutumba P., Sipsma H., Smallegange E., Macy R., de Jong J. (2014). School-based mental health intervention for children in war-affected Burundi: A cluster randomized trial. BMC Med..

[95] Tol W.A., Komproe I.H., Jordans M.J.D., Vallipuram A., Sipsma H., Sivayokan S., Macy R.D., De Jong J.T. (2012). Outcomes and moderators of a preventive school-based mental health intervention for children affected by war in Sri Lanka: A cluster randomized trial. World Psychiatry.

[96] Tol W.A., Komproe I.H., Susanty D., Jordans M.J.D., Macy R.D., De Jong J. (2008). School-based mental health intervention for children affected by political violence in Indonesia—A cluster randomized trial. JAMA.

[97] Tol W.A., Komproe I.H., Jordans M.J.D., Gross A.L., Susanty D., Macy R.D., de Jong J.T.V.M. (2010). Mediators and moderators of a psychosocial intervention for children affected by political violence. J. Consul. Clin. Psychol..

[98] Jordans M.J.D., Komproe I.H., Tol W.A., Susanty D., Vallipuram A., Ntamatumba P., Lasuba A.C., De Jong J.T.V.M. (2011). Practice-Driven Evaluation of a Multi-Layered Psychosocial Care Package for Children in Areas of Armed Conflict. Community Ment. Health Journal.

[99] Jordans M.J.D., Tol W.A., Komproe I.H. (2011). Mental health interventions for children in adversity: Pilot-testing a research strategy for treatment selection in low-income settings. Soc. Sci. Med..

[100] Jordans M.J., Tol W.A., Susanty D., Ntamatumba P., Luitel N.P., Komproe I.H., de Jong J.T. (2013). Implementation of a mental health care package for children in areas of armed conflict: A case study from Burundi, Indonesia, Nepal, Sri Lanka, and Sudan. PLoS Med.

[101] Layne C.M., Saltzman W.R., Poppleton L., Burlingame G.M., Pašalić A., Duraković E., Mušić M., Ćampara N., Dapo N., Arslanagić B. (2008). Effectiveness of a school-based group psychotherapy program for war-exposed adolescents: A randomized controlled trial. J. Am. Acad.f Child Adolesc. Psychiatry.

[102] Botha U.A., Koen L., Galal U., Jordaan E., Niehaus D.J.H. (2014). The rise of assertive community interventions in South Africa: A randomized control trial assessing the impact of a modified assertive intervention on readmission rates; a three year follow-up. BMC Psychiatry.

[103] Fayyad J.A., Farah L., Cassir Y., Salamoun M.M., Karam E.G. (2010). Dissemination of an evidence-based intervention to parents of children with behavioral problems in a developing country. Eur. Child Adolesc. Psychiatry.

[104] Singla D.R., Kumbakumba E., Aboud F.E. (2015). Effects of a parenting intervention to address maternal psychological wellbeing and child development and growth in rural Uganda: A community-based, cluster-randomised trial. Lancet Glob.Health.

[105] Singla D.R., Kumbakumba E. (2015). The development and implementation of a theory-informed, integrated mother-child intervention in rural Uganda. Soc. Sci. Med..

[106] Chatterjee S., Chowdhary N., Pednekar S., Cohen A., Andrew G., Araya R., Simon G., King M., Telles S., Verdeli H. (2008). Integrating evidence-based treatments for common mental disorders in routine primary care: Feasibility and acceptability of the MANAS intervention in Goa, India. World Psychiatry.

[107] Wu Z., Detels R., Zhang J., Li V., Li J. (2002). Community-Based Trial to Prevent Drug Use Among Youths in Yunnan, China. Am. J. Public Health.

[108] Bonhauser M., Fernandez G., Püschel K., Yañez F., Montero J., Thompson B., Coronado G. (2005). Improving physical fitness and emotional well-being in adolescents of low socioeconomic status in Chile: Results of a school-based controlled trial. Health Promot. Int..

[109] Hou Y., Hu P., Zhang Y., Lu Q., Wang D., Yin L., Chen Y., Zou X. (2014). Cognitive behavioral therapy in combination with systemic family therapy improves mild to moderate postpartum depression. Rev. Bras. Psiquiatr..

[110] Bolton P., Bass J., Betancourt T., Speelman L., Onyango G., Clougherty K.F., Neugebauer R., Murray L., Verdeli H. (2007). Interventions for depression symptoms among adolescent survivors of war and displacement in northern Uganda: A randomized controlled trial. JAMA.

[111] Chatterjee S., Leese M., Koschorke M., McCrone P., Naik S., John S., Dabholkar H., Goldsmith K., Balaji M., Varghese M. (2011). Collaborative community based care for people and their families living with schizophrenia in India: Protocol for a randomised controlled trial. Trials.

[112] Mutamba B.B., Kane J.C., De Jong J., Okello J., Musisi S., Kohrt B.A. (2018). Psychological treatments delivered by community health workers in low-resource government health systems: Effectiveness of group interpersonal psychotherapy for caregivers of children affected by Nodding Syndrome in Uganda. Psychol. Med..

[113] Lay B., Salize H.J., Dressing H., Rüsch N., Schönenberger T., Bühlmann M., Bleiker M., Lengler S., Korinth L., Rössler W. (2012). Preventing compulsory admission to psychiatric inpatient care through psycho-education and crisis focused monitoring. BMC Psychiatry.

[114] Shidhaye R., Murhar V., Gangale S., Aldridge L., Shastri R., Parikh R., Shrivastava R., Damle S., Raja T., Nadkarni A. (2017). The effect of VISHRAM, a grass-roots community-based mental health programme, on the treatment gap for depression in rural communities in India: A population-based study. Lancet Psychiatry.

[115] Maes K. (2016). The Lives of Community Health Workers: Local Labor and Globbal Health in Urban Ethiopia.

[116] Maes K., Kalofonos I. (2013). Becoming and remaining community health workers: Perspectives from Ethiopia and Mozambique. Soc. Sci. Med..

[117] Maes K., Closser S., Vorel E., Tesfaye Y. (2015). Using community health workers. Ann. Anthropol.Pract..

[118] Hanlon C., Tesfaye M., Wondimagegn D., Shibre T. (2010). Ethical and professional challenges in mental health care in low- and middle-income countries. Int. Rev. Psychiatry.

[119] Compton M.T., Bakeman R., Broussard B., Hankerson-Dyson D., Husbands L., Krishan S., Stewart-Hutto T., D’Orio B.M., Oliva J.R., Thompson N.J. (2013). The police-based crisis intervention team (CIT) model: II. effects on level of force and resolution, referral, and arrest. Psychiatr. Serv..

[120] Kohrt B.A., Blasingame E., Compton M.T., Dakana S.F., Dossen B., Lang F., Strode P., Cooper J. (2015). Adapting the Crisis Intervention Team (CIT) Model of Police–Mental Health Collaboration in a Low-Income, Post-Conflict Country: Curriculum Development in Liberia, West Africa. Am. J. Public Health.

[121] Zafar S., Sikander S., Hamdani S.U., Atif N., Akhtar P., Nazir H., Maselko J., Rahman A. (2016). The effectiveness of Technology-assisted Cascade Training and Supervision of community health workers in delivering the Thinking Healthy Program for perinatal depression in a post-conflict area of Pakistan – study protocol for a randomized controlled trial. Trials.

[122] Jordans M.J.D., Aldridge L., Luitel N.P., Baingana F., Kohrt B.A. (2017). Evaluation of outcomes for psychosis and epilepsy treatment delivered by primary health care workers in Nepal: A cohort study. Int. J. Ment. Health Syst..

[123] WHO (2014). Mental Health Atlas.

[124] Thornicroft G. (2012). Evidence-based mental health care and implementation science in low- and middle-income countries. Epidemiol. Psychiatr. Sci..

[125] NIMH Collaborative Hubs for International Research on Mental Health (U19). http://www.nimh.nih.gov/about/organization/gmh/globalhubs/index.shtml.

[126] Government of Canada Grand Challenges Canada: Global Mental Health. http://www.grandchallenges.ca/grand-challenges/global-mental-health/.

[127] Murray L.K.P., Skavenski S.M.S.W.M.P.H., Bass J.P., Wilcox H.P., Bolton P.M.M.P.H., Imasiku M.P., Mayeya J.M.P.H. (2014). Implementing Evidence-Based Mental Health Care in Low-Resource Settings: A Focus on Safety Planning Procedures. J. Cogn. Psychother..

[128] Murray L.K., Tol W., Jordans M., Sabir G., Amin A.M., Bolton P., Bass J., Bonilla-Escobar F.J., Thornicroft G. (2014). Dissemination and implementation of evidence based, mental health interventions in post conflict, low resource settings. Intervention.

[129] Semrau M., Lempp H., Keynejad R., Evans-Lacko S., Mugisha J., Raja S., Lamichhane J., Alem A., Thornicroft G., Hanlon C. (2016). Service user and caregiver involvement in mental health system strengthening in low- and middle-income countries: Systematic review. BMC Health Serv. Res..

[130] Lempp H., Abayneh S., Gurung D., Kola L., Abdulmalik J., Evans-Lacko S., Semrau M., Alem A., Thornicroft G., Hanlon C. (2018). Service user and caregiver involvement in mental health system strengthening in low- and middle-income countries: A cross-country qualitative study. Epidemiol. Psychiatr. Sci..

[131] Abayneh S., Lempp H., Alem A., Alemayehu D., Eshetu T., Lund C., Semrau M., Thornicroft G., Hanlon C. (2017). Service user involvement in mental health system strengthening in a rural African setting: Qualitative study. BMC Psychiatry.

[132] Gurung D., Upadhyaya N., Magar J., Giri N.P., Hanlon C., Jordans M.J.D. (2017). Service user and care giver involvement in mental health system strengthening in Nepal: A qualitative study on barriers and facilitating factors. Int. J. Ment. Health Syst..

[133] Rai S., Gurung D., Kaiser B.N., Sikkema K.J., Dhakal M., Bhardwaj A., Tergesen C., Kohrt B.A. (2018). A service user co-facilitated intervention to reduce mental illness stigma among primary healthcare workers: Utilizing perspectives of family members and caregivers. Fam. Syst. Health.

[134] Kohrt B.A., Jordans M.J.D., Turner E.L., Sikkema K.J., Luitel N.P., Rai S., Singla D.R., Lamichhane J., Lund C., Patel V. (2018). Reducing stigma among healthcare providers to improve mental health services (RESHAPE): Protocol for a pilot cluster randomized controlled trial of a stigma reduction intervention for training primary healthcare workers in Nepal. Pilot Feasibil. Stud..

[135] Collins P.Y., Musisi S., Frehywot S., Patel V. (2015). The core competencies for mental, neurological, and substance use disorder care in sub-Saharan Africa. Glob. Health Action.

[136] Padmavati R. (2012). Community mental health services for the mentally ill: Practices and ethics. Int. Rev. Psychiatry.

[137] Kohrt B.A., Ramaiya M.K., Rai S., Bhardwaj A., Jordans M.J.D. (2015). Development of a scoring system for non-specialist ratings of clinical competence in global mental health: A qualitative process evaluation of the Enhancing Assessment of Common Therapeutic Factors (ENACT) scale. Glob. Ment. Health.

[138] Kohrt B.A., Jordans M.J.D., Rai S., Shrestha P., Luitel N.P., Ramaiya M., Singla D., Patel V. (2015). Therapist Competence in Global Mental Health: Development of the Enhancing Assessment of Common Therapeutic Factors (ENACT) Rating Scale. Behav. Res. Ther..

[139] Singla D.R., Weobong B., Nadkarni A., Chowdhary N., Shinde S., Anand A., Fairburn C.G., Dimijdan S., Velleman R., Weiss H. (2014). Improving the scalability of psychological treatments in developing countries: An evaluation of peer-led therapy quality assessment in Goa, India. Behav. Res. Ther..

[140] Subba P., Luitel N.P., Kohrt B.A., Jordans M.J.D. (2017). Improving detection of mental health problems in community settings in Nepal: Development and pilot testing of the community informant detection tool. Conf. Health.

[141] Epping-Jordan J.E., Harris R., Brown F.L., Carswell K., Foley C., García-Moreno C., Kogan C., van Ommeren M. (2016). Self-Help Plus (SH+): A new WHO stress management package. World Psychiatry.

[142] van Ginneken N., Maheedhariah M.S., Ghani S., Ramakrishna J., Raja A., Patel V. (2017). Human resources and models of mental healthcare integration into primary and community care in India: Case studies of 72 programmes. PLoS ONE.

